# A loss of mature microglial markers without immune activation in schizophrenia

**DOI:** 10.1002/glia.23962

**Published:** 2021-01-07

**Authors:** Gijsje J. L. J. Snijders, Welmoed van Zuiden, Marjolein A. M. Sneeboer, Amber Berdenis van Berlekom, Astrid T. van der Geest, Tatiana Schnieder, Donald J. MacIntyre, Elly M. Hol, René S. Kahn, Lot D. de Witte

**Affiliations:** ^1^ Department of Psychiatry, University Medical Center Utrecht Brain Center, Utrecht University, Brain Center Rudolf Magnus University Medical Center Utrecht, Utrecht University (BCRM‐UMCU‐UU) Utrecht The Netherlands; ^2^ Department of Psychiatry Icahn School of Medicine New York New York USA; ^3^ Department of Translational Neuroscience (BCRM‐UMCU‐UU) Utrecht The Netherlands; ^4^ Department of Psychiatry Columbia University New York New York USA; ^5^ Division of Psychiatry, Centre for Clinical Brain Sciences University of Edinburgh Edinburgh UK; ^6^ Neuroimmunology, Netherlands Institute for Neuroscience, An Institute of the Royal Academy of Arts and Sciences Amsterdam The Netherlands; ^7^ Mental Illness Research, Education and Clinical Center (MIRECC), James J Peters VA Medical Center Bronx New York USA

**Keywords:** gene expression, immunology, microglia, postmortem, schizophrenia

## Abstract

Microglia, the immune cells of the brain, are important for neurodevelopment and have been hypothesized to play a role in the pathogenesis of schizophrenia (SCZ). Although previous postmortem studies pointed toward presence of microglial activation, this view has been challenged by more recent hypothesis‐driven and hypothesis‐free analyses. The aim of the present study is to further understand the observed microglial changes in SCZ. We first performed a detailed meta‐analysis on studies that analyzed microglial cell density, microglial morphology, and expression of microglial‐specific markers. We then further explored findings from the temporal cortex by performing immunostainings and qPCRs on an additional dataset. A random effect meta‐analysis showed that the density of microglial cells was unaltered in SCZ (ES: 0.144 95% CI: 0.102 to 0.390, *p* = .250), and clear changes in microglial morphology were also absent. The expression of several microglial specific genes, such as *CX3CR1*, *CSF1R*, *IRF8*, *OLR1*, and *TMEM119* was decreased in SCZ (ES: −0.417 95% CI: −0.417 to −0.546, *p* < .0001), consistent with genome‐wide transcriptome meta‐analysis results. These results indicate a change in microglial phenotype rather than density, which was validated with the use of TMEM119/Iba1 immunostainings on temporal cortex of a separate cohort. Changes in microglial gene expression were overlapping between SCZ and other psychiatric disorders, but largely opposite from changes reported in Alzheimer's disease. This distinct microglial phenotype provides a crucial molecular hallmark for future research into the role of microglia in SCZ and other psychiatric disorders.

## Main Points

- Microglia density is unaltered in postmortem brain tissue of schizophrenia patients, but several mature microglial markers are downregulated in schizophrenia. This expression pattern is largely opposite from microglial changes in Alzheimer's disease.

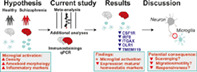

## 1 INTRODUCTION

Genetic and epidemiological studies have suggested a causal role for the immune system in schizophrenia (SCZ) pathogenesis (Benros et al., 2014; Pouget et al., 2019; Ripke et al., 2014; Sekar et al., 2016; van Mierlo, Schot, Boks, & de Witte, 2019). This is supported by changes in immune‐related markers reported in blood, cerebrospinal fluid, and brain tissue of SCZ patients (Gandal et al., 2018; Upthegrove, Manzanares‐Teson, & Barnes, 2014; Wang & Miller, 2018). It has, therefore been hypothesized that microglia, the immune cells of the brain, play an important role in SCZ (Howes & McCutcheon, 2017). Microglia are part of the innate immune system and regulate inflammatory responses in the central nervous system (CNS) (Tambuyzer, Ponsaerts, & Nouwen, 2009). Microglia also control synaptic pruning, a process thought to be dysregulated in SCZ (Berdenis van Berlekom et al., 2019). Synaptic pruning is required for normal brain development and is mediated through the complement cascade (Stephan, Barres, & Stevens, 2012). Copy number variants in complement factor 4A (C4A) are associated with SCZ (Sekar et al., 2016), and increased synaptic pruning was observed in patient‐derived microglial cell cultures (Sellgren et al., 2019).

Many brain disorders coincide with microglial activation, a state characterized by an increased cell density, and a morphological shift from ramified toward amoeboid, and upregulated expression of several inflammatory molecules (Kettenmann, Hanisch, Noda, & Verkhratsky, 2011). A previous review and independent meta‐analysis (Trépanier, Hopperton, Mizrahi, Mechawar, & Bazinet, 2016; Van Kesteren et al., 2017) suggested an activated microglia phenotype in postmortem brain tissue of patients with SCZ. However, these reviews also noted substantial heterogeneity between studies, and a generally limited number of subjects, ranging from *N* = 5–35. This heterogeneity is probably caused by a large variety of pre‐ and postmortem confounders, methodological differences, and analysis of divergent brain regions (Powchik et al., 1998). In addition, samples that partially overlapped between the studies were included in the meta‐analysis. In contrast, three recent genome‐wide transcriptomic studies on SCZ postmortem brain tissue, with sample sizes ranging from *N* = 159–559 patients (Bergon et al., 2015; Gandal et al., 2018; Gandal, Zhang, et al., 2018), did not find increased expression of most of these microglial markers. Instead, the expression of several well‐known microglial genes, such as *CX3CR1*, *TMEM119*, *TREM2*, *CSF1R*, *ITGAM*, *CD86*, and *OLR1*, were downregulated in one or more of these studies.

The goal of the present study was to better understand these reported microglial changes in schizophrenia. Our aim was to provide an up‐to‐date overview of hypothesis‐driven studies that analyzed microglial density, morphology, and expression of microglial‐specific genes. We performed a structural review followed by a qualitative and quantitative assessment of the selected studies. We included an additional dataset using postmortem samples from the Netherlands (NBB) and Edinburgh Brain Banks (EBB) to explore previous significant microglial findings in the temporal cortex (Van Kesteren et al., 2017). In addition, we further elucidate changes in mRNA expression identified by the aforementioned hypothesis‐free transcriptomic studies on bulk brain tissue (Gandal, Zhang, et al., 2018). By applying a recently published core microglial signature (Patir, Shih, McColl, & Freeman, 2019) on this dataset we identified up‐ and downregulated genes that are likely to reflect changes in microglia. We analyzed how these microglia‐related changes overlap with gene expression changes in other disorders, such as autism spectrum disorder (ASD), bipolar disorder (BPD), and Alzheimer's disease (AD). Altogether, this study provides an overview of microglial changes in postmortem brain tissue of patients with SCZ.

## 2 MATERIAL AND METHODS

### 2.1 Meta‐analysis

#### 2.1.1 Search strategy

The quantitative review was performed according to the Preferred Reporting Items for Systematic Reviews and Meta‐Analyses (PRISMA) (Liberati et al., 2009). We performed two separate systematic searches for articles analyzing microglial density and morphology changes or changes in microglial‐specific gene expression. We used PubMed and EMBASE, and checked references of included studies and previous reviews (Suzuki et al., 2019;Trépanier et al., 2016; Van Kesteren et al., 2017). We used the following search terms: (a) (postmortem OR postmortem OR autopsy) AND (schizophreni* OR psychotic OR psychosis) AND (microgli* OR macrophage*); (b) (postmortem OR postmortem OR autopsy) AND (AIF1 OR IBA1 OR Iba‐1 OR CD68 OR NOX2 OR CYBB OR HLADR* OR HLA‐DR* OR P2RY12 OR P2Y12 OR TMEM119 OR CX3CR1 OR ITGB2 OR ITGAX OR CD11c OR GPR34 OR CSF1R OR IRF8 OR TGFB1 OR TREM2 OR DAP12 OR TYROBP OR OLR1) AND (schizophreni* OR psychotic OR psychosis). The microglia markers (*n* = 17) were selected because they were used as microglia markers in studies that came out of our first search and recent reviews (Trépanier et al., 2016; Van Kesteren et al., 2017), or that have been more recently described as microglia‐specific markers (Bennett et al., 2016; Butovsky et al., 2014). Furthermore, these markers are specifically expressed in microglia compared to other cells in the CNS and are all part of a defined microglial signature recently described by (Patir et al., 2019). We excluded transcriptomic studies (micro‐array and RNA sequencing) from the meta‐analysis, since we assumed a high chance of selection/reporting bias, as only significant results were reported in most of these studies. However, to validate our meta‐analysis findings, we extracted results from the largest transcriptomic study in SCZ to date, which includes data on all detected genes (Gandal, Zhang, et al., 2018). The search cut‐off date was February 28, 2020. Screening and selection of studies were performed independently by three authors (GS, WZ, and LW). Disagreements were resolved through discussion.

#### 2.1.2 Inclusion criteria

Pre‐specified inclusion criteria were: (a) human postmortem studies; comparing (b) patients with a diagnosis of SCZ with (c) healthy controls; (d) measuring microglial density or gene expression of microglial markers; (e) original research, published in a peer‐reviewed journal; and (f) written in English.

#### 2.1.3 Qualitative assessment

Several criteria were assessed to evaluate the quality of the included studies. Study design was rated for the following aspects: blinding researchers to diagnostic or clinical information, neuropathological assessment, the degree of matching of control and patient population, and whether correction of general (age/PMI) and other confounding factors, such as medication use, pH, cause of death, illness duration, age of onset, was applied. Methodology was assessed for complete description of technical methods and subsequent analyses. For reporting, studies were evaluated for whether important details regarding psychopathologic examination, population demographics, and main outcome variables could be retrieved (Tables S5 and S11).

#### 2.1.4 Data extraction

When data records in the original article were not given or insufficient to generate effect sizes, corresponding authors were asked to provide raw data. In the case of follow‐up data, included outcomes of the largest sample size were included. If data were not reported numerically in the manuscript, data was extracted using https://automeris.io/WebPlotDigitizer/. The following variables were extracted for potential moderator analyses: sample size, methods, brain bank, brain region and area (cortical or subcortical), age, sex, postmortem interval (PMI), and pH (Tables S6, S7, S8, S12 and S13).

#### 2.1.5 Statistical approach meta‐analysis

Meta‐analyses were carried out using the Comprehensive Meta‐Analysis (CMA) software developed by Biostat (Borenstein, Hedges, Higgins, & Rothstein, 2009). Change in microglial number or microglial gene expression per brain (sub)region was used to quantify effect size (ES) between SCZ and controls. We used sample size, mean, and standard deviation (SD) to generate ES. Hedges's *g* and the upper/lower limit of the 95% CI were used to express ES. Given the heterogeneity among studies, we used a random effects model for all comparisons (Higgins, Thompson, & Spiegelhalter, 2009). The Cochran's *Q*‐statistic test, displaying a chi‐square distribution with *k* − 1 degrees of freedom, was performed to evaluate the existence of heterogeneity. High *Q*‐values indicate that the variability among studies is higher than would be expected due to randomness, and further examination of subgroups is warranted. *I*‐squared (*I* ^2^) was calculated to estimate the amount of heterogeneity. *I* ^2^ reflects which proportion of the observed variance reflects differences in true effect size rather than sampling error (range 0–100%) (Higgins, 2003). Potential outlier studies were defined as those with standardized residual *z*‐scores of effect sizes exceeding ±1.96 (*p* ≤ .05 two tailed). The potential for publication bias was assessed by visual examination of Funnel plots and by the Egger's test (which was considered significant if the one‐sided *p*‐value was ≤.10) (Egger, Smith, Schneider, & Minder, 1997). Random‐effects meta‐regression analyses were performed to analyze the role of potential confounding factors (age, sex, PMI, pH). An exploratory subgroup analysis was performed on biological variables (brain regions, technical variation (methods, outcome measures) to assess sources of heterogeneity. We grouped outcomes for analyzing different brain regions in four subgroups: frontal cortex (including the dorsal, ventral, and dorsolateral prefrontal cortex, orbitofrontal cortex, midfrontal cortex, entorhinal cortex), temporal cortex, occipital cortex and limbic system (including the mid(anterior) cingulate cortex, hippocampus, amygdala, thalamus, dorsal raphe nucleus, basal ganglia). A common among‐study variance was assumed across different subgroups, and within‐group estimates of tau‐squared were pooled. Between‐group differences were tested using the *Q*‐test based analysis of variance to determine whether the variance within subgroups was significantly smaller than the variance of all the combined data (Qbetween = Qtotal − (QSubgroup_A_ + QSubgroup_B_). Cell density and gene expression studies were analyzed separately. For the microglial cell density studies, each study included only one marker (or a double staining) as outcome measurement, but various studies included multiple brain regions. Microglial cell density and gene expression measurements in different brain regions within the same cohort/study are not independent of each other. To prevent overrepresentation of the results from studies analyzing multiple brain regions in our analysis across all brain regions, we therefore used a conservative approach and nested data by computing combined scores from all measurements across different brain regions within one study. Forest plots of unnested data are depicted in the Figures of Supporting Information.

### 2.2 SCZ cohort

#### 2.2.1 Human brain tissue

Paraffin‐embedded and frozen postmortem samples of the superior temporal gyrus were provided by the Netherlands (Rademaker, de Lange, & Palmen, 2018) and Edinburgh Brain Banks (Millar et al., 2007) (NBB and EBB). The permission to collect human brain material was obtained from the Ethical Committee of the VU University Medical Center, Amsterdam, The Netherlands, and the East of Scotland Research Ethics Service REC1. Permission for brain autopsy and the use of brain tissue and accompanying clinical information for research purposes was obtained per donor ante‐mortem. Control donors were defined as donors without a clinical diagnosis of a major depressive disorder, bipolar disorder, or psychotic disorder according to the DSM‐IV, DSM‐III‐R, or DSM‐III. If the DSM classification was not available, the DSM characterization was based on retrospective medical chart review by two independent psychiatrists. Cases clinically diagnosed with Alzheimer's or Parkinson's disease, Amyotrophic Lateral Sclerosis, Multi System Atrophy, or brain diseases caused by an infection (meningitis and encephalitis) were excluded. There was no significant difference between controls and patients in age, sex, postmortem interval (PMI), or pH (Table S1). Detailed clinicopathological information per donor is provided in Table S2.

#### 2.2.2 Iba1 immunohistochemistry

Microglial density and morphology were characterized by ionized calcium‐binding adapter molecule 1 (Iba1) immunohistochemistry as described before (Sneeboer et al., 2019). Paraffin‐embedded tissue of the superior temporal gyrus was sectioned at 7 μm for SCZ patients (*N* = 12) and controls (*N* = 16). The sections were deparaffinized using a standard xylene and alcohol series, followed by blocking of endogenous peroxidase with PBS, 1% H_2_O_2_ (Merck, Germany). For antigen retrieval, sections were heated in 0.01 mM citrate buffer (Merck, Darmstadt, Germany), 0.05% Tween‐20 (Merck, Darmstadt, Germany), pH = 6.0 for 15 min. Subsequently, nonspecific binding was blocked in PBS with 1% normal horse serum (NHS, Thermo Fisher Scientific, MA), 0.1% bovine serum albumin (BSA, Merck, Darmstadt, Germany), and 0.2% Triton X (Merck, Darmstadt, Germany). Sections were subsequently incubated with a rabbit polyclonal anti‐Iba1 antibody (Wako Pure Chemical Industries, Ltd., 1:1000) at 4°C. Next day, secondary goat‐anti‐rabbit biotin (Jackson ImmunoResearch Laboratories, Inc., 1:400) was added, followed by avidin‐biotin‐peroxidase (AB) complex (Vector Laboratories). To visualize the microglia, the sections were incubated with a 3,3′ diaminobenzidine (DAB) substrate (DAKO). Finally, tissue sections were dehydrated using an alcohol and xylene series and embedded in Entellan (Merck, Darmstadt, Germany). Iba1‐DAB stainings were visualized with a ZEISS Imager M2 light microscope. Per tissue section, six pictures of 615 x 450 μm were randomly taken throughout the cortex blinded for diagnosis. A qualitative assessment was done of the microglial morphology in each picture, as previously described by (Torres‐Platas, Cruceanu, Chen, Turecki, & Mechawar, 2014), classifying them into a ramified, primed, reactive or amoeboid category. Open‐source software ImageJ was used to quantify microglial cell numbers and Iba1 positive  area covered. A particle analysis macro‐script was used to count microglial cell numbers of the DAB staining. The macro consisted of the following steps: transformation to 8‐bit; scaling of pixels to μm (according to microscope guidelines); automated default threshold application (minimum radius mask of 1 and maximum radius mask of 3); conversion to mask; particle filtration with size > 80; option “outline” and “exclude cells on the edge”. Thereafter, the average number of microglial cells of six pictures was calculated and divided by the area of the pictures. This method was previously validated (Sneeboer et al., 2019). The macro for the Iba1 positive area covered consisted of the following steps: transformation to 8‐bit; scaling of pixels to μm (according to microscope guidelines); automated default threshold application; conversion to mask; particle filtration with size > 0.01, option “outline”, “exclude cells at edge” and “summarize”. The pictures were manually viewed, pictures with poor performance were excluded, and the Iba1 positive‐area covered was averaged for the 3–6 non‐excluded pictures.

#### 2.2.3 Iba1/TMEM119 immunofluorescence

Immunofluorescence double staining with Iba1 and Transmembrane Protein 119 (TMEM119) antibodies was performed by sectioning paraffin‐embedded tissue of the superior temporal gyrus at 7 μm for SCZ patients (*N* = 18) and controls (*N* = 19). After deparaffinization, blocking of endogenous peroxidase, antigen retrieval, and blocking of nonspecific binding with normal horse serum, sections were incubated with a polyclonal goat anti‐Iba1 antibody Ab5076 (Abcam) and a polyclonal rabbit anti‐TMEM119 antibody HPA051870 (Atlas Antibodies). This was followed by staining with donkey anti‐rabbit Alexa Fluor 488 and donkey anti‐goat Cy3 antibodies (Jackson Immunoresearch Laboratories), as well as Hoechst (Thermo Scientific). Images (615 × 450 μm) of six randomly selected areas throughout the grey matter of the cortex were collected with a Zeiss Axio Scope A1 fluorescence microscope. Most cells were positive for both Iba1 and TMEM119. While we only rarely observed cells that were TMEM119^+^ and Iba1^−^, we found a variable degree of Iba1^+^/TMEM119^−^ across donors. To analyze case–control differences, we calculated the percentage of TMEM119^+^/Iba1^+^ by dividing the number of TMEM119^+^/Iba1^+^ cells by the total number of Iba1^+^ cells, both manually counted.

#### 2.2.4 Gene expression analysis

Five 50 μm sections of grey matter of the superior temporal gyrus were dissected manually from cryosections for SCZ patients (*N* = 9) and controls (*N* = 14). RNA was isolated using Trizol reagent (Thermo Fisher Scientific, MA), followed by addition of 100 μl chloroform and centrifugation at 12,000 rcf at 7°C for 15 min. Subsequently, RNA was precipitated from 100–200 μl of the aqueous top phase by mixing with an equal volume of isopropanol, using 1 μl glycogen as a carrier, and stored overnight at −20°C. The next day, samples were centrifuged at maximum speed at 4°C for 60 min. The pellet was washed twice with 75% ETOH, air‐dried, and dissolved in 8 μl Milli‐Q water. Concentrations of extracted RNAs were measured using a nanodrop (ND‐1000; NanoDrop Technologies, Rockland, DE). RNA was reversed transcribed using a Quantitect Reverse Transcription kit (QIAGEN, Hilden, Germany) according to the manufacturer's protocol. RNA supplemented with Milli‐Q water to a volume of 6 μl was mixed with 1 μl gDNA Wipe‐Out buffer and incubated at 42°C for 2 min. After addition of the master mix containing 2 μl Reverse‐Transcriptase buffer, 0.5 μl Reverse‐Transcriptase enzyme, and 0.5 μl RT random hexamers primer mix, samples were incubated at 42°C for 30 min, followed by 95°C for 3 min. Samples were subsequently stored at −20°C until further use. Quantitative real‐time polymerase chain reaction (qPCR) was performed on a QuantStudio™ 6 Flex Real‐Time PCR System (Life Technologies Corporation, NY). Per reaction, 3.5 ng input of cDNA was mixed with Milli‐Q water, 5 μl SYBRgreen PCR Master Mix (Roche; Life Technologies Corporation, Grand Island, NY), and 1 μl primer mix (2 pmol/mL; see Table S3) until a final volume of 11 μl. All primers were intron‐spanning and designed with the online tool of NCBI. The following cycle conditions were used: 50°C for 2 min, 95°C for 10 min, 40 cycles at 95°C of 15 s, and at 60°C for 60 s. Gene expression was normalized to reference genes (Glyceraldehyde 3‐phosphate dehydrogenase (GAPDH), β‐actin (ACTB), and Succinate Dehydrogenase Complex Flavoprotein Subunit A (SDHA)) according to the ΔΔCT method. These genes were selected from a larger set of housekeeping genes based on the Vandesompele method (Vandesompele et al., 2002). Gene expression values were compared with other gene expression studies by dividing the mean ΔCT scores of SCZ patients by the mean of the controls and applying a log2 transformation. The relative gene expression was expressed as a log2 fold change (log2FC) value.

#### 2.2.5 Statistics

Statistical analysis was performed with SPSS IBM 25. Assumption of normality was tested by visual checks using histograms and by the Shapiro Wilk test. Mann–Whitney *U* tests were applied to test the differences between patients and controls. Spearman rank correlation tests were used to determine the relation between age, PMI, pH, sex, and microglial density (Iba1^+^‐cells/mm^2^ and total Iba1^+^ ‐area covered) and the microglial gene expression analysis. Subsequently, an ANCOVA analysis was performed to study the interaction effects of these covariates on microglial density and microglial gene expression. In the case of non‐normally distributed data, log transformation was used. Results were adjusted for multiple testing by dividing the probability level of *p* < .05 by the number of performed tests.

### 2.3 Cross‐disorder comparison of microglial gene expression changes

We used a recently identified core signature of *N* = 249 genes that are specifically expressed by microglia compared to other CNS cell‐types (Patir et al., 2019). We extracted differential gene expression data of these genes (log2 fold changes) from the largest bulk brain tissue transcriptome studies on SCZ (Gandal, Zhang, et al., 2018), BD (Gandal, Zhang, et al., 2018), ASD (Gandal, Zhang, et al., 2018), and AD (Friedman et al., 2018). Correlations between log2 fold changes in different conditions and the significance of these correlations were calculated with the R function *rcorr* in R package *Hmisc* (Harrell, 2020). Overlaps between up‐ and downregulated genes in SCZ versus the other disorders were determined and tested for significance using the R function *fisher*.*test* (Harrell, 2020).

## 3 RESULTS

### 3.1 Literature search microglial density and morphology studies

After title and abstract screening for inclusion and exclusion criteria, 38 studies were selected for full‐text assessment. Twenty‐two studies were excluded (Table S4), including two studies (Busse et al., 2012; Steiner et al., 2006) containing samples that overlapped with other studies (Gos et al., 2014; Steiner et al., 2008). The study with the largest sample size was included. The database searches in PubMed and EMBASE, and additional records identified via cross‐referencing, yielded a total of 16 records (Figure S1) that were all included in the qualitative assessment (Table S5). Authors of six studies (Comte, Kotagiri, & Szele, 2012; Connor, Guo, & Akbarian, 2009; Fillman et al., 2013; Foster et al., 2006; Schnieder et al., 2014; Seredenina et al., 2017) were contacted for additional information, with additional data received from two studies (Fillman et al., 2013; Schnieder et al., 2014). A total of 12 studies were included in the microglia density meta‐analysis, with a total of 238 patients and 252 controls (Table S6). These studies encompassed 12 different brain regions. Most studies analyzed microglial density and a few studies also investigated changes in microglial morphology. Various markers were used to visualize microglia: HLA‐DR, Iba1, CD68, and NOX2. NOX2 was used in only one study (Seredenina et al., 2017) (Table S7). HLA‐DR and CD68 are upregulated on activated microglia, whereas Iba1 is more stably expressed. NOX2 has not been used before for assessing microglia density, but the authors show that the protein is expressed on microglia, and this was confirmed by the study of Patir (Patir et al., 2019). An overview of the included studies and extracted data can be found in Tables S5‐8.

### 3.2 Quality assessment

A quality assessment was performed for all studies assessing methodology, study design, and reporting. The overall quality of the studies was rated high. Most studies ruled out neuropathology, described their applied methods extensively, performed case–control matching, and reported important confounders (age/PMI/sex). Correction for these confounders was also often applied. As expected, more recent studies scored higher on these quality measures than some of the initial studies. As microglia activation can be caused by a variety of reasons, a full description of demographics can be helpful. However, this was present in only half of the studies.

### 3.3 Review and meta‐analysis of microglial density

**FIGURE 1:**
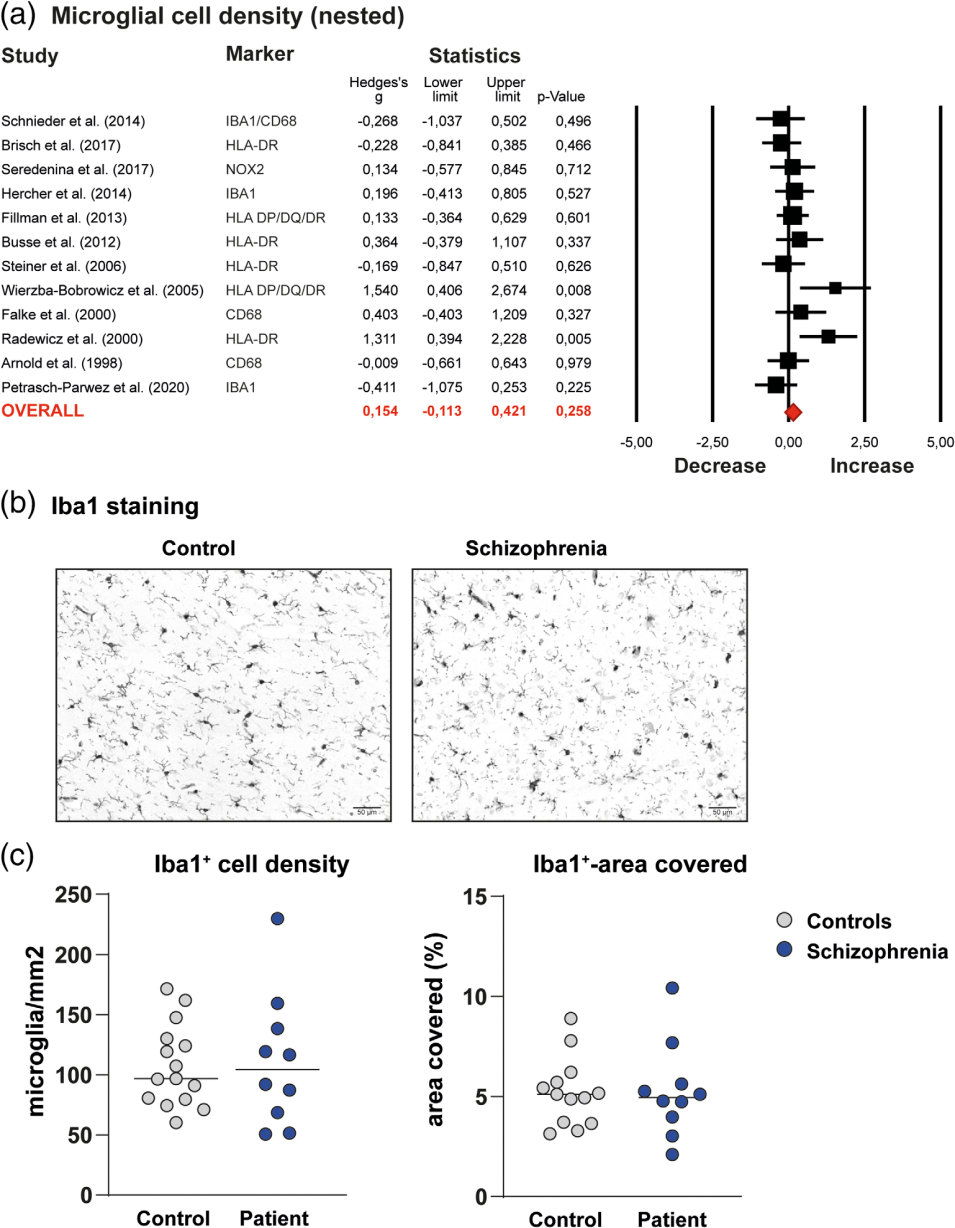
Meta‐analysis microglial cell density and newly generated microglial cell density data in the temporal cortex of patients with schizophrenia and controls from the Netherlands Brain Bank (NBB) and Edinburgh Brain Bank (EBB). (a) Forest plot of primary meta‐analysis of studies assessing microglial cell density in schizophrenia (SCZ) postmortem brain tissue. Data from different regions in one study were nested to prevent overrepresentation. The forest plots in both graphs show the data included in a random effects meta‐analysis, representing effect sizes (Hedges's *g*′) with 95 confidence interval (CI) for differences between patients with SCZ and controls in each study. The Square size is proportional to study weight. Diamonds at the bottom of the graphs reflect the pooled effect sizes. (b) Representative pictures of the microglial staining of the temporal cortex of patients with schizophrenia (SCZ) and controls using immunohistochemistry with antibodies to ionized calcium‐binding adaptor molecule (Iba1). (c) The density of microglia (microglia/mm^2^) and percentage of the area covered with Iba1^+^ staining were quantified automatically using imageJ. Grey dots represent the controls (*N* = 16), blue dots the patients (*N* = 12), horizontal lines the median expression [Color figure can be viewed at wileyonlinelibrary.com]

A random‐effects meta‐analysis was performed on 12 studies to analyze differences in microglial density irrespective of brain region. Estimated ES for different brain regions within one study was nested to prevent overrepresentation. Figure 1a showed no significant differences in microglial density between SCZ patients and controls (ES: 0.154 95% CI −0.113‐0.421, *p* = .258). Unnested data displayed similar results (ES: 0.183 95% CI: −0.000 to 0.365 *p* = .05; Figure S2). The heterogeneity for the studies was low to moderate (*I* ^2^ = 18.9%), and the variability between studies was not significant (*Q* = 18.4, *p* = .06). Inspection of the funnel plot and the Egger's test showed indication for publication bias (*p* = .01) (Figure S4). Two studies were defined as potential outlier studies, exceeding residual z‐scores of 1.96 (Radewicz, Garey, Gentleman, & Reynolds, 2000; Wierzba‐Bobrowicz, Lewandowska, Lechowicz, Stepień, & Pasennik, 2005)). Sensitivity analysis, excluding these outlier studies, indicated no significant differences between SCZ and controls in microglia cell density (ES: 0.010 95% CI: −0.197 to 0.217). A meta‐regression analysis checked for potential confounder variables (age, gender, pH, and PMI) showed that none of these variables had a significant effect on the outcome (*p* > .05; data not shown). An exploratory subgroup analysis was performed to assess possible sources of variation. No significant differences were observed in microglia density between different microglia markers (HLA‐DR, CD68, Iba1, NOX2), automated or manual counting methods, or cortical or subcortical studies (data not shown). When segregating the analysis per brain regions, we found a significant increase in microglial density in the temporal cortex (ES: 2.27 95% CI: 1.469–3.090, *p* < .001, Q‐between = 29.72; *p* < .001) in SCZ patients, but this was not observed in the other brain regions (frontal and occipital cortex or limbic system; Figure S3). However, the two temporal cortex studies were identified as potential outliers, exceeding residual *z*‐scores of 1.96 (Radewicz et al., 2000; Wierzba‐Bobrowicz et al., 2005).

### 3.4 Review of microglial morphology

Table S8 provides an overview of studies that assessed the morphology of microglia in SCZ. Three studies did not observe morphological changes (Brisch et al., 2017; Petrasch‐Parwez et al., 2020; Schnieder et al., 2014). Three studies reported morphological differences between patients and controls, but the direction of results was different: Wierzba et al. reported an increased density of both ramified and amoeboid cells (Wierzba‐Bobrowicz et al., 2005); Hercher et al. reported the presence of cells with an amoeboid morphology in white matter in 3/20 SCZ patients but none of the controls (Hercher, Chopra, & Beasley, 2014); while Radewicz et al. reported that microglia tended to have more ramified processes in SCZ (Radewicz et al., 2000).

### 3.5 Microglial density and morphology NBB and EBB

To further explore previous findings in the temporal cortex, microglial density and morphology were assessed in a new postmortem brain tissue SCZ cohort using immunostainings for microglial marker Iba1 (Figure 1b,c). Morphological assessment did not show microglia differences between patients and controls (Table S9). We quantified microglial density using two parameters: Iba1‐covered area and Iba1^+^ cells/mm^2^. The correlation between these parameters was 0.57 (*p* = .008). The number of Iba1^+^ cells/mm^2^ and total Iba1‐covered area were not different from controls (Iba1^+^‐cells/mm^2^: SCZ: 111.3 ± 34.1; control 107.4 ± 55.0, *p* = .82; Iba1‐covered area: SCZ: 5.28 ± 2.35; control 5.23 ± 1.69, *p* = .47). Age, PMI and pH were associated with microglial density and Iba1‐covered area. Controlling for these covariates using an ANCOVA did not change this result (Iba1^+^‐cells/mm^2^: *F*_1,14_ = 0.10, *p*‐value = .74, partial *ŋ* ^2^ = 0.008; total Iba1‐covered area: *F*_1,14_ = 0.35, *p*‐value = .57, partial *ŋ* ^2^ = 0.03). The current study was included in the meta‐analysis described before (Figures S10–S12). Overall, the results did not change and microglial density did not differ between SCZ patients and controls (ES: 0.144 95% CI: 0.102–0.390, *p* = .250; Figure S10). When analyzing the different brain regions including the current study, we still found a significant increase in microglial density in the temporal cortex compared to the other brain regions, albeit the effect size was smaller (ES: 1.256 95% CI: 0.55–1.96, *p* < .001, *Q*_between_ = 16.4, *p* < .001; Figure S12).

### 3.6 Microglial gene expression studies

Following our systematic search for gene expression of microglial markers, 85 records were found in PubMed and EMBASE, from which seven studies were finally included (Figure S5; Table S10). An overview of quality assessment, included studies in meta‐analysis, and extracted data can be found in Tables S11–S13. A total of 255 patients and 261 controls were analyzed (Table S12). These studies encompassed four different brain regions (frontal including DLPFC), temporal, cingulate cortex, and hippocampus) (Table S13).

### 3.7 Review and meta‐analysis of expression microglial markers

**FIGURE 2:**
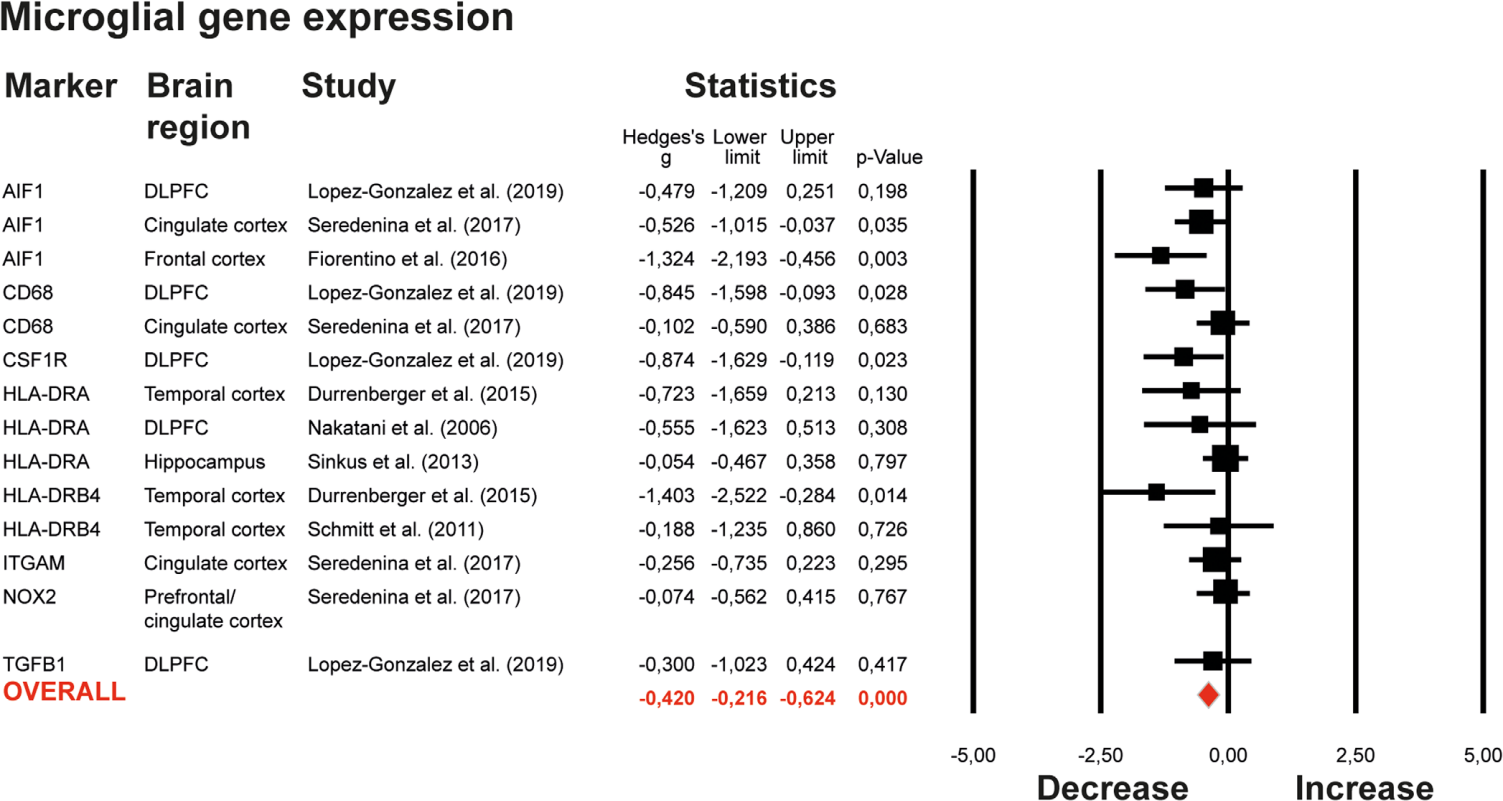
Meta‐analysis on expression of microglial genes. Forest plot of primary meta‐analysis on expression of microglial genes. Studies are ordered by the microglial gene that was studied. The column “Brain region” refers to the brain region studied (DLPFC‐Dorsolateral Prefrontal Cortex; ACC‐Anterior Cingulate Cortex; mACC‐mid‐Anterior Cingulate Cortex). Data from different regions in one study were nested to prevent overrepresentation. The forest plots in both graphs show the data included in a random effects meta‐analysis, representing effect sizes (Hedges's *g*′) with 95 confidence interval (CI) for differences between patients with SCZ and controls in each study. The Square size is proportional to study weight. Diamonds at the bottom of the graphs reflect the pooled effect sizes [Color figure can be viewed at wileyonlinelibrary.com]

A random‐effect meta‐analysis revealed a significant decrease in gene expression of microglial markers for unnested outcomes (ES: −0.420 95% CI −0.216 to −0.624, *p* < .0001, Figure 2). Exploratory subgroup analyses revealed a significant decreased expression of microglial genes in cortical tissues (ES: −0.553 95% CI −0.817 to −0.290, *p* < .0001), but no change in subcortical tissues (ES: −0.212 95% CI −0.454 to 0.029, *p* = .08; *Q*_between_ = 3.49; *p* = .06; Figure S6). Further zooming in on brain regions, a significant decrease in temporal (ES: −0.744 95% CI: −1.361 to −0.128, *p* = .018) and frontal cortex (ES: −0.541 95% CI: 0.810 to −0.218, *p* = .001) was observed, but no change in regions related to the limbic system (ES: −0.213 95% CI: −0.461 to 0.035, *p* = .092; *Q*_between_ = 3.87, *p* = .14; Figure S7). We found a significant decrease for the marker *AIF1* (ES: −0.694 95% CI: −1.168 to −0.220, *p* < .004), but not for *HLA‐DR* (*Q*_between_ = 4.53; *p* = .60, Figure S8). The heterogeneity was low to moderate (*I* ^2^ = 26.7%). The *Q*‐value indicated no significant variability between studies (*Q* = 19.1, *p* = .162). The funnel plot and Egger's test indicated the presence of publication bias (*p* = .001; Figure S9). One study was defined as a potential outlier (Durrenberger et al., 2015). Sensitivity analysis, excluding this potential outlier study, did not change the results (ES: −0.319 95% CI −0.490 to −0.148, *p* < .0001). None of the potential moderators (gender, age, PH, PMI and brain bank) were associated with the ES or significantly changed heterogeneity (data not shown).

### 3.8 Microglial gene expression NBB and EBB

**FIGURE 3:**
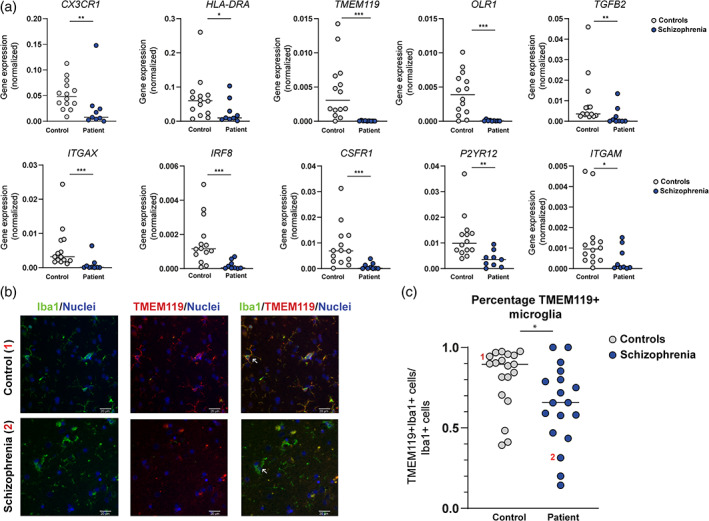
Expression of a panel of microglial genes in the temporal cortex of patients with schizophrenia and controls from the Netherlands Brain Bank and Edinburgh Brain Bank. (a) mRNA expression levels of a panel of microglial genes in controls (*N* = 14, grey dots) and patients with schizophrenia (SCZ; *N* = 9, blue dots) as determined in the temporal cortex by qPCR. Horizontal lines show median expression. Non‐parametric testing was applied. *p < .05, **p < .010, ***p < .001. (b) Paraffin sections of temporal cortex of controls (*N* = 19) and patients with SCZ (*N* = 18) were stained with antibodies to Iba1 (green) and TMEM119 (red), and nuclei visualized using Hoechst (blue). Representative pictures for the two groups are shown: White arrow in control represents positive staining for TMEM119/Iba1, white arrow in in schizophrenia represents positive staining for Iba1, but negative staining for TMEM119. (c) The quantification of the percentage of TMEM119^+^/Iba1^+^ of the total population of Iba1^+^. Number 1 and 2 refer to the donors that are also shown in the stainings of Figure 3b. Horizontal lines show median expression. Non‐parametric testing was applied. *p < .05 [Color figure can be viewed at wileyonlinelibrary.com]

mRNA expression was determined for a panel of 16 microglia‐specific markers in the temporal cortex of SCZ patients and controls (Table 1). *CSF1R*, *IFR8*, *ITGAX*, *OLR1*, and *TMEM119* were significantly downregulated. *CX3CR1*, *HLA‐DRA*, *ITGAM*, *ITGB2*, and *P2RY12* were also downregulated in SCZ patients (Figure 3a), but these genes did not survive correction for multiple testing. Age, sex, and PMI were associated with microglial gene expression. However, controlling for age, sex, and PMI using an ANCOVA did not change the results, except *ITGAM* lost significance after correction (*F*_1,18_ = 2.12, *p*‐value = .162, partial *ŋ* ^2^ = 0.106). No significant differences were observed for *AIF1*, *CD68*, *GPR34*, *ITGB2*, *TGFB2*, *TREM2*, *TYROBP* between SCZ patients and controls. We included our novel data of the NBB/EBB cohort in our meta‐analysis of published studies (Figure S13–S16). These analyses also showed a significant decrease in gene expression of core microglial genes (ES: −0.417 95% CI: −0.417 to −0.546, *p* < .0001; Figure S13). To further validate these findings, we compared our qPCR results with results of the most recent transcriptome meta‐analysis (Gandal, Zhang, et al., 2018) (Table 1). *CSF1R*, *OLR1*, *IRF8*, *CX3CR1*, *HLA‐DR*, *ITGAM*, *ITGAX*, *P2RY12*, and *TMEM119* were significantly downregulated in both studies. Altogether, we found an unaltered density of Iba1^+^ cells, but decreased gene expression of several microglial markers. These results could be an indication of an altered overall microglial phenotype with decreased expression of several markers. However, recent studies have shown that microglia are a heterogeneous cell population that consists of clusters with different characteristics (Grabert et al., 2016; Masuda, Sankowski, Staszewski, & Prinz, 2020; Mathys et al., 2017; Prinz, Priller, Sisodia, & Ransohoff, 2011; Van Wageningen et al., 2019). The separation between some of these clusters and macrophages is challenging. Our results could, therefore, also reflect shifts in subclusters of microglia/myeloid cells. To further explore these two possibilities at the protein level, we performed double‐labeled immunostainings with Iba1 and TMEM119 antibodies (Figure 3b). Co‐localization analysis showed that SCZ patients had significantly less microglia that were positive for both Iba1 and TMEM119 in the temporal cortex (*p* = .027) (Figure 3c), which indicates a shift in subsets of microglia/myeloid cells. We observed donor‐donor differences in the morphology of TMEM119^+^ cells, but did not find an association with disease status.

**TABLE 1:** qRT‐PCR measured expression of 16 microglia‐specific genes in postmortem temporal cortex brain tissue lysates of schizophrenia patients compared to healthy controls. Samples derived from the Netherlands Brain Bank and Edinburgh Brain Bank are compared to gene expression results extracted from the genome‐wide RNA‐sequencing study performed by the PsychENCODE consortium described by Gandal, Zhang, et al. (2018). Genes that showed significant changes with a similar direction of effect in the current study and Gandal et al. are highlighted in bold

Gene	Mean (*SD*) controls NBB and EBB	Mean (*SD*) SCZ NBB and EBB	Log_2_FC NBB and EBB	Log_2_FC (Gandal, Zhang, et al., 2018)
*AIF1*	0.032 (0.027)	0.044 (0.040)	0.454	−0.192**
*CD68*	0.009 (0.011)	0.005 (0.005)	−0.989	−0.065
***CSF1R***	**0.009 (0.008)**	**0.001 (0.001)**	**−3.306** **	**−0.176** **
*CX3CR1*	0.052 (0.029)	0.026 (0.047)	−0.956*	−0.464**
*GPR34*	0.001 (0.006)	0.014 (0.030)	0.553	−0.216**
*HLA‐DRA*	0.067 (0.063)	0.028 (0.035)	−1.279*	−0.129*
***IRF8***	**0.004 (0.003)**	**0.002 (0.002)**	**−2.945** **	**−0.229** **
*ITGAM*	0.001 (0.001)	0.0004 (0.0005)	−1.618*	−0.166**
***ITGAX***	**0.006 (0.006)**	**0.001 (0.002)**	**−2.535** **	**−0.305** **
*ITGB2*	0.011 (0.009)	0.003 (0.005)	−1.894*	−0.095
***OLR1***	**0.004 (0.003)**	**8.908E‐05 (9.606E‐05)**	**−5.500** **	**−0.215** **
*P2RY12*	0.012 (0.008)	0.003 (0.003)	−1.716*	−0.416**
*TGFB1*	0.007 (0.005)	0.009 (0.009)	0.379	0.066*
***TMEM119***	**0.004 (0.004)**	**4.843E‐05 (4.165E‐05)**	**−6.532** **	**−0.237** **
*TREM2*	0.003 (0.003)	0.003 (0.004)	−0.169	−0.245**
*TYROBP*	0.016 (0.014)	0.034 (0.035)	1.108	−0.132*

*Note*: Genes marked in bold are significantly downregulated in SCZ patients in our dataset and Gandal et al. after correction for multiple testing (Gandal, Zhang, et al., 2018).

Abbreviations: EBB, Edinburgh Brain Bank; Log_2_FC, Log2 fold change; NBB, Netherlands Brain Bank; SCZ, schizophrenia.

* *p*‐value < .05;

** Adjusted *p*‐value < .05.

### 3.9 Comparing the expression levels of microglia‐specific genes in bulk tissue across disorders

**FIGURE 4:**
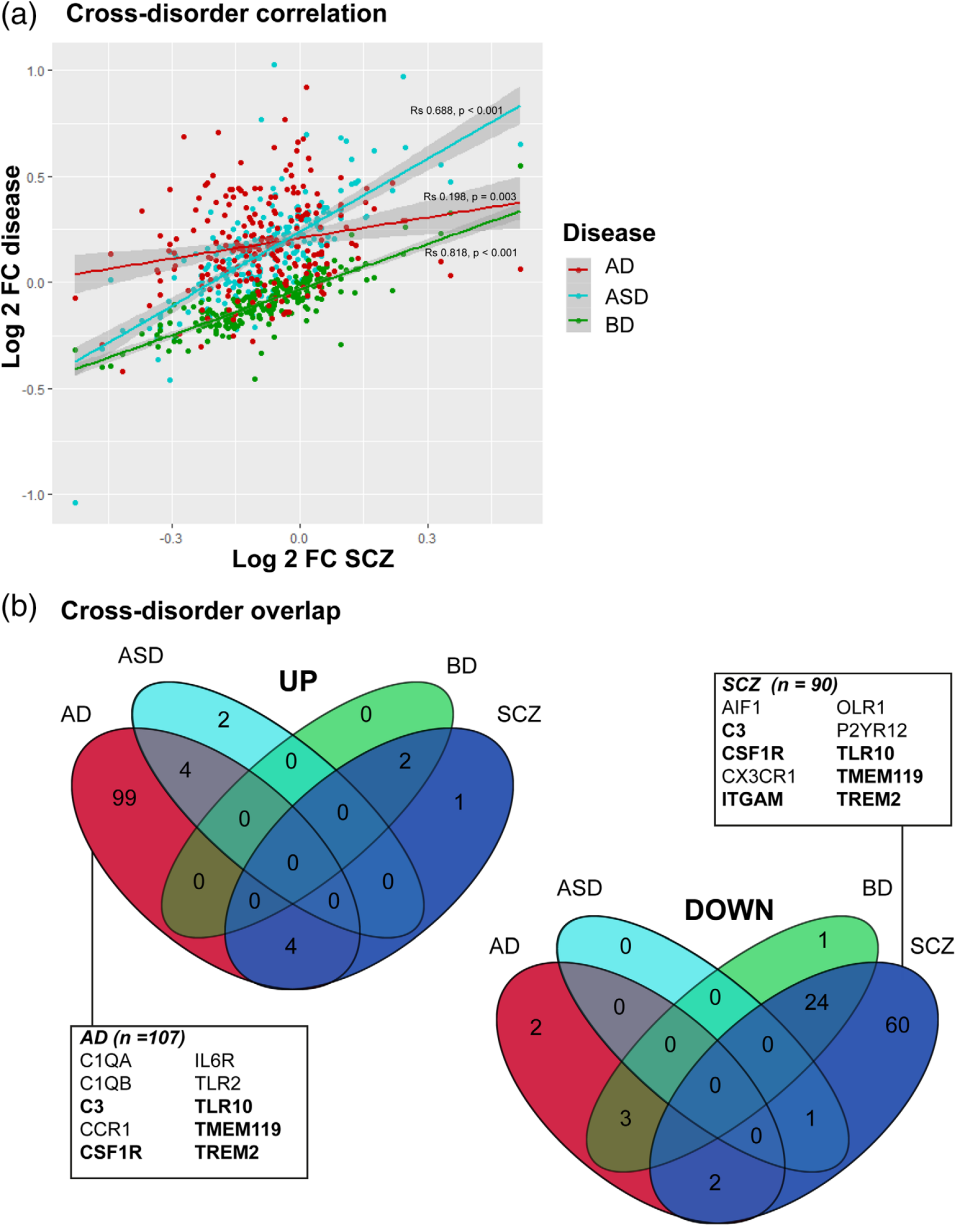
Cross‐disorder analysis. (a) Comparison between transcriptional changes of a set of microglial signature genes (*N* = 243) in bulk brain tissue across diseases. Log2 fold changes of 243 core microglial genes assessed in cortex of patients with schizophrenia (SCZ) (Gandal, Zhang, et al., 2018), plotted against the log2 fold changes reported in cortex of patients with Alzheimer's Disease (AD) (Friedman et al., 2018), Autism Spectrum Disorder (ASD) and Bipolar Disorder (BD) (Gandal, Zhang, et al., 2018). Spearman correlation and p‐values are provided. (b) Overlaps between the number of microglia signature genes up‐ or downregulated in SCZ, ASD, AD, and BD. Well‐known microglial markers are highlighted; genes in **bold** are significantly upregulated in AD, but downregulated in SCZ [Color figure can be viewed at wileyonlinelibrary.com]

To further understand how the phenotype of microglia is changed in SCZ, a set of microglia‐specific genes (*N* = 243) (Patir et al., 2019) and available results from the aforementioned transcriptome study (Gandal, Zhang, et al., 2018) were analyzed. In SCZ, 90/243 microglia‐specific genes are downregulated, and 7 are upregulated (Table S14). Downregulated genes include *C3*, *P2RY12*, *P2RY13*, *CSF1R*, *CX3CR1*, *TMEM119*, *ITGAM*, *ITGAX*, *OLFML3*. A cross‐disorder analysis demonstrated that expression changes of these microglia‐specific genes are correlated between SCZ and ASD or BD (Figure 4a). Interestingly, most of these 243 microglia‐specific genes were upregulated in AD, but downregulated in SCZ (Figure 4b). Thirty‐seven genes showed an opposite effect in these disorders, being upregulated in AD and downregulated in SCZ. These included *CD33*, *TREM2*, *TMEM119*, *CSF1R*, and *TLR10*.

## 4 DISCUSSION

The aim of the present study was to provide an update of the microglial changes that have been found in postmortem brain tissue of patients with SCZ. We systematically investigated published studies assessing microglial cell density, morphology and expression of microglial genes in postmortem brain tissue of SCZ patients and performed additional experiments on temporal cortex of a separate and new cohort.

### 4.1 Microglial density and morphology in SCZ

Previous reviews by Trepanier and Van Kesteren reported evidence for an increased microglial density in SCZ (Trépanier et al., 2016; Van Kesteren et al., 2017). Here, we included 10 additional studies (Brisch et al., 2017; Durrenberger et al., 2015; Fiorentino et al., 2016; López‐González et al., 2019; Nakatani et al., 2006; Petrasch‐Parwez et al., 2020; Schmitt et al., 2011; Schnieder et al., 2014; Seredenina et al., 2017; Sinkus, Adams, Logel, Freedman, & Leonard, 2013) and excluded two studies with overlapping samples (Gos et al., 2014; Steiner et al., 2008), as well as one study that had a strong effect in the previous reviews (ES: 1.79 95% CI 0.76–2.83), but was retracted because of data falsification (Rao, Kim, Harry, Rapoport, & Reese, 2013). In addition, we applied a nesting approach on studies including multiple brain regions. The heterogeneity of our updated meta‐analysis was lower compared to the previous study (Van Kesteren et al., 2017), and we no longer found a significant difference in microglial density in SCZ. A significantly increased microglial density was only found in the temporal cortex. However, that result was based on two smaller studies (*N* = 18 and *N* = 17 in total) (Radewicz et al., 2000; Wierzba‐Bobrowicz et al., 2005) that were identified as potential outliers with methodological limitations, as reported in the qualitative assessment. In addition, we were not able to validate an increased density in the temporal cortex of an additional cohort, with the limitation that the sample size of our additional cohort was also limited (*N* = 28 in total). Although we found several studies reporting on morphological changes, the results were inconsistent, which is probably related to the large variety of read‐outs that were used on studies with small sample sizes and the challenges in quantifying morphological assessments.

### 4.2 Microglial gene expression in SCZ

The previous reviews reported indications for neuroinflammation. In both studies, the authors included mRNA and protein expression of a variety of inflammatory markers (Trépanier et al., 2016; Van Kesteren et al., 2017), such as IL‐6, IL‐1β, and IL‐8, which are not specific to microglia, but are also expressed in astrocytes and other cell types in the brain. In addition, the results were heterogeneous and except for an increased expression of *IL6*, increased expression of these markers was not found in the most recent and robust transcriptome meta‐analysis (Gandal, Zhang, et al., 2018). Since we were specifically interested in microglia, we only included expression of a set of microglial‐specific genes (Patir et al., 2019). Our qPCR analysis, our meta‐analysis, and the most recent genome‐wide transcriptomic analysis all show a consistent decreased expression of several microglial genes, including the well‐known markers *HLA‐DRA* and *ITGAM*. It would be interesting to understand how the observed findings relate to the binding of the TSPO tracer. Positron emission tomography studies with TSPO tracers have been often used to analyze neuroinflammation or microglial activation in vivo. TSPO PET studies in schizophrenia patients have shown heterogeneous results. While the earlier studies reported increased binding of the tracers, more recent studies did not find this effect or even a lower binding (Marques et al., 2019; Plavén‐Sigray et al., 2018). Moreover, there is a debate about what type of changes in glial cells are reflected by changes in TSPO binding (Notter, Coughlin, Sawa, & Meyer, 2018; Owen et al., 2017; Sneeboer et al., 2020). Our current study illustrates that microglial changes go beyond the activated/non‐activated dichotomy. The microglia signature that we describe here may provide novel molecular imaging targets.

### 4.3 Translation of gene expression changes to cellular changes

In our meta‐analysis and additional immunostainings, we did not find indications for changes in microglial density. In our gene expression analyses, we found decreased expression of several microglia‐specific genes, including *P2RY12*, *CSF1R*, and *TMEM119*. To further explore these findings at the cellular level, we performed a staining with Iba1 and TMEM119 and found a decreased percentage of Iba1 cells expressing TMEM119. TMEM119 has recently been identified as a marker that is highly specific to microglia (Bennett et al., 2016; Satoh et al., 2016). In contrast to Iba1, which is also expressed on infiltrating macrophages (Imai, Ibata, Ito, Ohsawa, & Kohsaka, 1996; Ohsawa, Imai, Kanazawa, Sasaki, & Kohsaka, 2000), it is exclusively expressed on microglia. Although the function of TMEM119 is still unknown, it has been identified as a downstream target of PU.1, a protein that is involved in microglia development (Satoh et al., 2016). TMEM119 is highly expressed on subclusters of microglial cells with more homeostatic properties, and this subcluster is partly lost with increasing age and in neurodegeneration as observed in Alzheimer's disease and amyotrophic lateral sclerosis brain tissue (Chiu et al., 2013; Holtman et al., 2015; Krasemann et al., 2017; Olah et al., 2018; Orre et al., 2014). These results suggest that microglia in schizophrenia do not all show the same changes in phenotype, but that there is a change in the composition of the microglia population in schizophrenia, similar to what has been shown for neurodegenerative disorders (Srinivasan et al., 2020). In addition, it is important to note that Iba1 is a marker that is often used as a marker for microglia, but that this marker is not restricted to microglia and also expressed at lower levels on for instance, infiltrating macrophages (Imai et al., 1996; Ohsawa et al., 2000). We performed a systematic search for the macrophage markers ITGA4, CD44, CD169, CD38, CD11a, PDL1 (see Figure S17) (Bowman et al., 2016; Greter, Lelios, & Croxford, 2015; Gu et al., 2016; Mrdjen et al., 2018), but we did not find any relevant articles. In addition, we extracted the data of these markers from the largest transcriptome meta‐analysis to date (Gandal, Haney, et al., 2018; Gandal, Zhang, et al., 2018). Except for a decreased expression of CD44, we found no indications for SCZ‐associated changes in infiltrating macrophages (Table S15). Analyses at the single‐cell level, such as mass cytometry (Böttcher et al., 2019; Sneeboer et al., 2019; Snijders et al., 2020) or single nuclei RNA‐seq (Masuda et al., 2020; Mathys et al., 2019) are needed to further understand how the composition of myeloid cells is changed in schizophrenia.

### 4.4 Interpretation of the identified SCZ‐associated microglial gene expression changes

**FIGURE 5:**
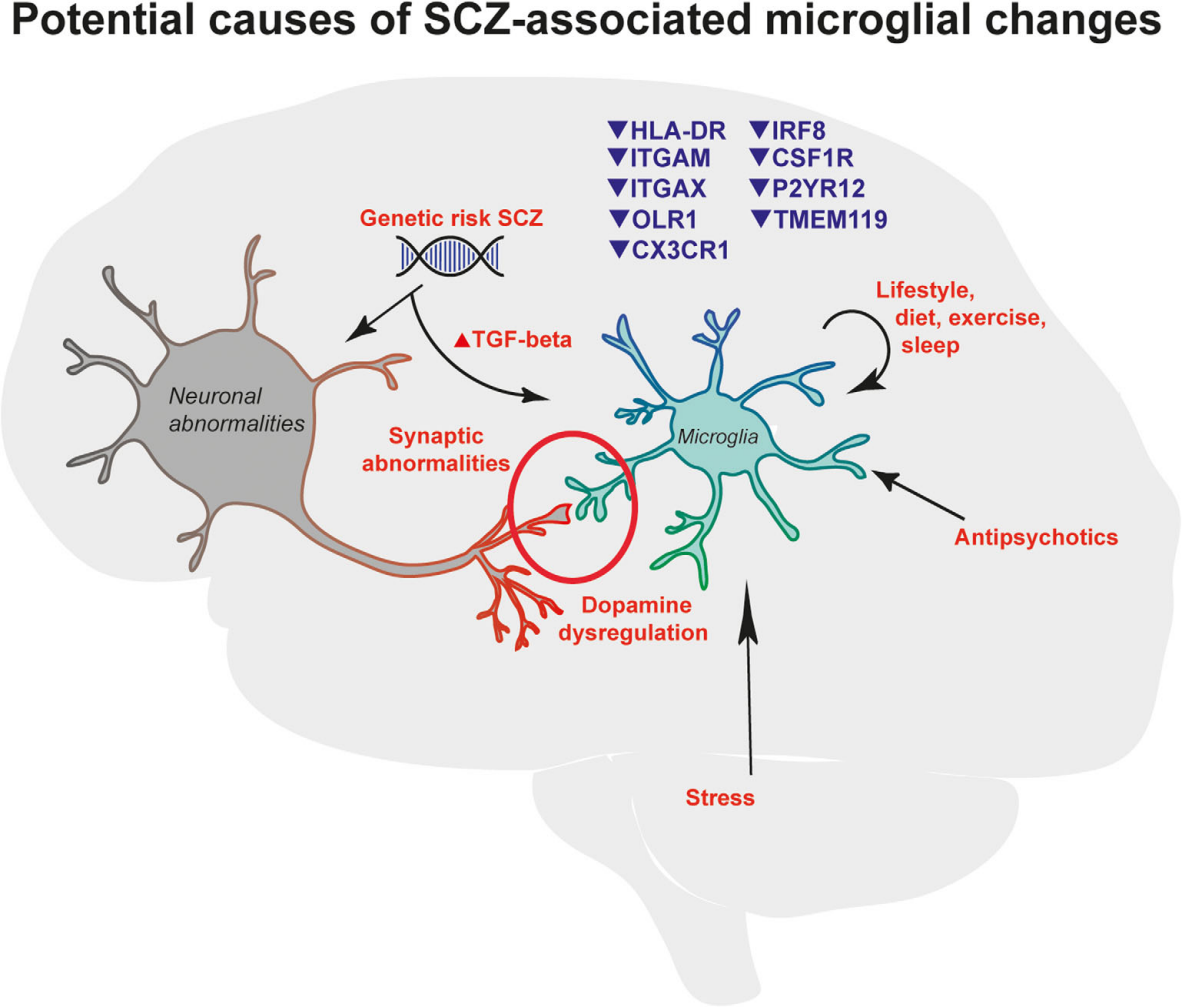
Schematic representation of potential causes of the observed microglia signature in SCZ. This figure summarizes the results from our study and suggests how these findings may interact and cause (or contribute) to SCZ [Color figure can be viewed at wileyonlinelibrary.com]

Here we describe a panel of various microglial‐specific genes that are consistently downregulated in SCZ. These changes showed similarities with other psychiatric disorders, but a distinct profile from AD. An important limitation for this cross‐disorder analysis, however, is that the AD data were derived from a separate transcriptome analysis (Friedman et al., 2018) rather than the combined analysis of different psychiatric disorders (Gandal, Zhang, et al., 2018). Many factors can potentially be involved in causing this SCZ‐related microglial profile, characterized by a loss of mature characteristics but without signs of overt immune activation. We have summarized some of the potential candidates in Figure 5. This includes a response of microglia to stress, a well‐known risk factor for developing psychiatric disorders (Zorn et al., 2017). Animal studies revealed that stress can induce a downregulation of similar microglial markers as observed in SCZ postmortem brain tissue, such as *CX3CR1*, *P2RY12*, and *TGFBR1* (Park et al., 2019). This microglial phenotype may also be a consequence of disrupted TGF‐β signaling, which is important for inducing and maintaining the distinct profile of microglia (Butovsky et al., 2014; Gosselin et al., 2017). The changes we observed showed a high resemblance with changes induced by culturing microglia after isolating them from postmortem brain tissue. However, these changes are counteracted when the cells are supplemented with TGF‐β (Gosselin et al., 2017). An overrepresentation of the TGF‐β signaling pathway in SCZ risk genes further supports this hypothesis (Chavarría‐Siles et al., 2007; Frydecka et al., 2013). Upstream from those gene expression changes might be epigenetic changes previously described for SCZ. The largest methylation study to date (Jaffe et al., 2015) found differential methylation of several transcription factors that are related to microglia development and phenotype, including MAFB, FOS, RUNX1, and MEF2C (Gosselin et al., 2017; Jaffe et al., 2015; van Mierlo et al., 2019). In addition to these potential causal pathways, this microglial signature may have also been triggered by any of the consequences of having SCZ, such as the use of medication (A.Bloomfield et al., 2018; Cotel et al., 2015; Kato et al., 2011) or an altered lifestyle since unhealthy diet, less physical activity and sleep, and obesity can all cause changes in microglia (Ingiosi, Opp, & Krueger, 2013; Jamatia et al., 2018; Valero, Paris, & Sierra, 2016).

**FIGURE 6:**
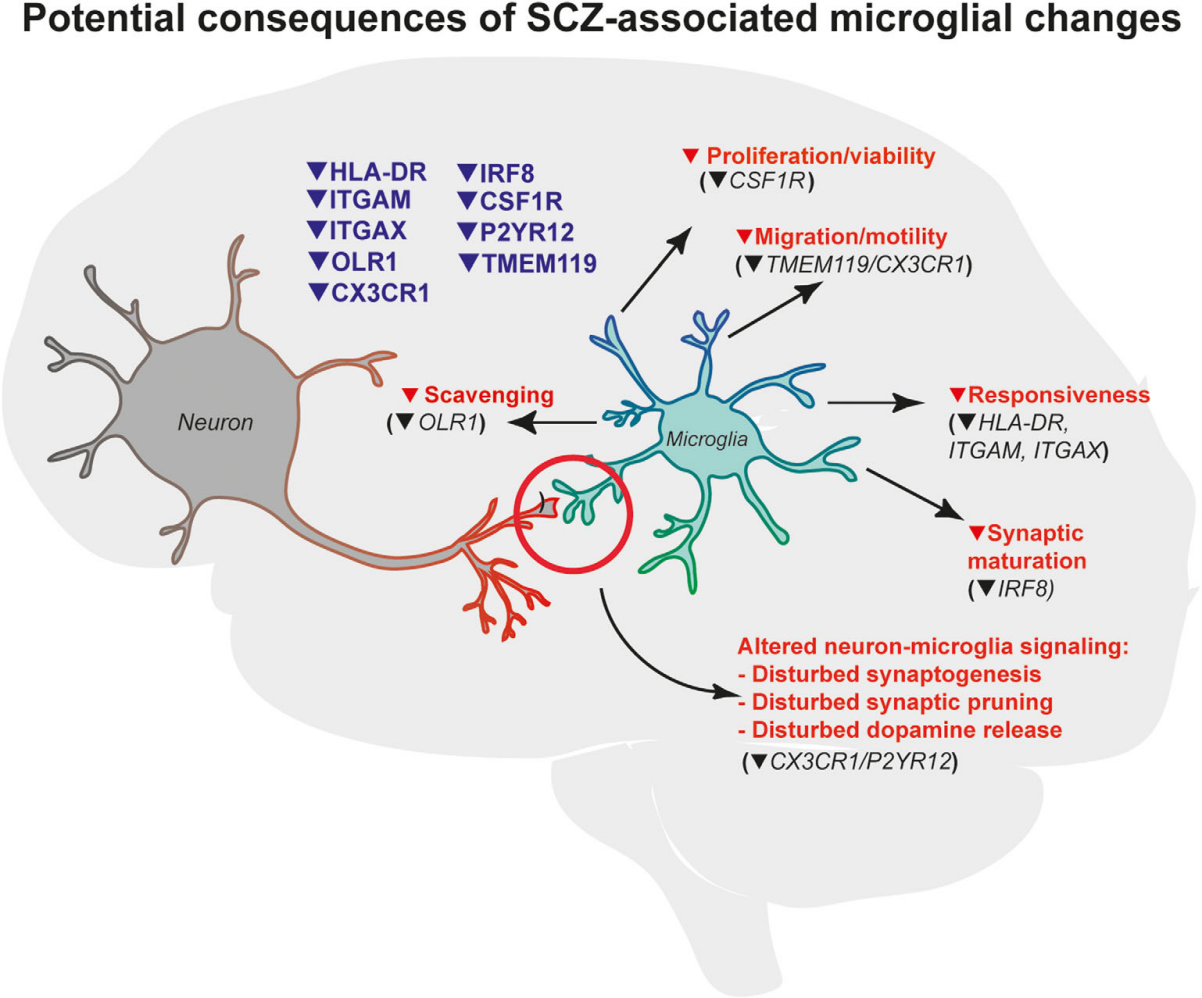
Schematic representation of potential consequences of the observed microglial signature in SCZ. This figure summarizes the results from our study and suggests how these findings may influence functions of microglia in SCZ [Color figure can be viewed at wileyonlinelibrary.com]

Whether the changes that we observed are involved in causing the disease is not known yet. In Figure 6 we speculate how the observed microglial changes could potentially contribute to the pathogenesis of SCZ. This includes dysregulation of the normal functions of microglia in neuroplasticity and neurotransmission by affecting the crosstalk between microglia, neurons, and astrocytes (Cserép et al., 2020). For instance, mice with *CX3CR1* deletion exhibit reduced dendritic spine pruning, abnormal synapse maturation, decreased functional connectivity, and behavioral abnormalities (Paolicelli et al., 2011). Recently, increased phagocytosis and increased inflammation were observed in a cellular model that deleted the *CX3CR1* gene in microglia derived from human induced pluripotent stem cells (hiPSC), suggesting that CX3CR1 in human microglia may contribute to microglial homeostasis by regulating inflammatory response and phagocytosis (Murai et al., 2020). Abnormalities in synaptic maturation, synapse elimination and spine density are thought to underlie SCZ, but also contribute to other psychiatric disorders, including ASD and BD (Gandal, Zhang, et al., 2018; Penzes, Cahill, Jones, Vanleeuwen, & Woolfrey, 2011). In addition, these microglial changes could also be involved in dopamine dysregulation in SCZ (Jaaro‐Peled, Ayhan, Pletnikov, & Sawa, 2010). *P2RY12* and related genes have been shown to play an important role in dopamine signaling (Krügel, Kittner, Franke, & Illes, 2001; Trendelenburg & Bültmann, 2000; Zhang, Yamashita, Ohshita, Sawamoto, & Nakamura, 1995). Our findings stress the need for more research to understand how changes in the molecular phenotype of microglia are related to changes in microglia–neuron interactions and how this influences brain development and function.

### 4.5 Strengths and limitations

Evidence for inflammation in postmortem brain tissue of SCZ patients has been reviewed before (Trépanier et al., 2016; Van Kesteren et al., 2017), but these studies combined different types of outcomes into one analysis. This can be problematic, since gene expression, protein expression and cell density do not necessarily correlate (Liu, Beyer, & Aebersold, 2016). In this study, we used a more conservative approach by separating density measures from gene expression outcomes. In addition, we prevented the overrepresentation of particular populations by excluding studies with overlapping samples. Subgroup analysis outcomes were performed on a limited number of studies and may have been susceptible to the influence of an outlier. Our comparative analysis may provide direction in the search for underlying mechanisms through the identification of circumstances similar and different to SCZ. However, the data on SCZ we used for comparison originated from bulk brain tissue sequencing, and it recently became clear that microglia‐specific changes are underrepresented in bulk data (Mathys et al., 2019). Although we selected genes that are strongly enriched in microglia, expression by other cell types could have contributed to the outcome. Thus, the exact microglia‐specific changes in SCZ remain to be determined. In addition, many factors, such as neuroleptics (Kowalski, Labuzek, & Herman, 2003) and use of second‐generation antipsychotics (Kato et al., 2011; Bloomfield et al., 2018; Cotel et al., 2015), cause of death, suicide attempts, somatic comorbidity (Mathys et al., 2019; Mizee et al., 2017; van der Poel et al., 2019), chronicity (Bitanihirwe & Woo, 2020), positive or negative symptomatology (Volk, 2017), age and sex may confound the results. We were not able to fully adjust for all these potential confounding effects in additional analyses due to small sample sizes in our own cohort and limited reporting across studies in the meta‐analysis.

### 4.6 Conclusions

Altogether we have found evidence for the presence of a distinct microglia phenotype in SCZ with potential overlap with other psychiatric disorders. The microglial gene expression changes identified in bulk brain tissue of SCZ patients need to be confirmed through sequencing of pure microglia populations by either isolating them from non‐frozen brain tissue (Melief et al., 2016) or using nuclei‐based techniques (Nott et al., 2019). Single‐cell/nuclei techniques (Masuda et al., 2020) will also uncover whether these changes are reflecting changes in the entire microglial population or just a subpopulation. The impact of these SCZ‐associated transcriptomic alterations on microglial function and, ultimately, the influence on brain development and function is important for understanding the relevance of these findings and will rely on studies in cell‐culture and animal models.

## CONFLICT OF INTEREST

All authors reported no financial, biomedical, or potential conflict of interest to declare.

## Supporting information


**Appendix** **S1**. Supporting Information.

**Appendix** **S2**. Figures.

**Appendix** **S3**. Tables.

## Data Availability

Data is available upon request.
