# Supplementary material for: A loss of mature microglial markers without immune activation in schizophrenia
Source: Glia. 2021 Jan 7;69(5):1251–67. doi: 10.1002/glia.23962 (PMC7986895; doi:10.1002/glia.23962)
Supplement: Supplementary file 1 — Appendix S1. Supporting Information. [file GLIA-69-1251-s002.docx]

**Legends Fig S1-S17

Supplementary Figure 1. Flowchart.** Study selection for cellular microglia studies. *The overlapping studies were (Gos et al., 2014) and (Busse et al., 2012), and (Steiner et al., 2006) and (Steiner et al., 2008) respectively. The studies with largest sample sizes were included.

**Supplementary Figure 2**. **Meta-analysis unnested microglial density data.** The forest plot shows all individual data points included in a random effects meta-analysis, representing effect sizes (Hedges’s g’) with 95 confidence interval (CI) for differences between SCZ patients and controls in each study. Square size is proportional to study weight. The column “Brain region” refers to the brain region studied (DLPFC-Dorsolateral Prefrontal Cortex; ACC-Anterior Cingulate Cortex; mACC-mid anterior Cingulate Cortex) and measurement (outcome given for a particular subgroup, such as activated or ramified cellular state). The column “marker” shows the marker that was used to assess microglial density in each study. The blue diamond represents the pooled effect of all separate data points.

**Supplementary Figure 3. Meta-analysis microglial cell density stratified per brain region.**
Forest plots meta-analyses for microglial density in schizophrenia (SCZ), stratified per brain region (frontal cortex, regions related to the limbic system, occipital cortex, and temporal cortex). The column “marker” shows the marker that was used to assess microglial density in each study. Diamonds reflect the pooled effect sizes.

**Supplementary Figure 4**. **Funnel plot cell density studies.**Meta-analysis funnel plot of studies included in primary meta-analysis of cellular microglia density in SCZ. Effect sizes (Hedges’s g) against Standard Error of each study. Asymmetry of the distribution within the funnel is used to determine the presence of publication bias. Red asterisks indicate right-skewed studies (Radewicz et al., 2000; Wierzba-Bobrowicz et al., 2005).

**Supplementary Figure 5. Flowchart.** Study selection for molecular marker microglia studies.

**Supplementary Figure 6. Subgroup meta-analysis gene expression levels in cortical or subcortical regions.**
Subgroup meta-analyses for molecular markers in SCZ stratified per cortex versus subcortical brain region. The column “marker” shows the marker that was used to assess microglial density in each study. Diamonds reflect the pooled effect sizes.

**Supplementary Figure 7. Meta-analysis gene expression levels stratified per brain region.**
Forest plot meta-analyses of expression levels of microglia genes, stratified per brain region (frontal cortex, regions related to the limbic system, and temporal cortex). The column “marker” shows the marker that was used to assess microglial density in each study. Diamonds reflect the pooled effect sizes.

**Supplementary Figure 8. Subgroup meta-analysis for microglia gene expression studies stratified per marker**. The column “Brain region” refers to the specific brain region studied (DLPFC-Dorsolateral Prefrontal Cortex). Diamonds reflect the pooled effect sizes.

**Supplementary Figure 9. Funnel plot microglia gene expression studies.**
Meta-analysis funnel plot of molecular markers measured in SCZ. Effect sizes (Hedges’s g) against Standard Error of each study. Asymmetry of the distribution within the funnel is used to determine the presence of publication bias. A red asterisk indicates left-skewed study (Durrenberger et al., 2015).

**Supplementary Figure 10.** **Meta-analysis cell density including newly generated data from the NBB and EBB.**  The forest plot shows nested data included in a random effects meta-analysis, representing effect sizes (Hedges’s g’) with 95 confidence interval (CI) for differences between SCZ patients and controls in each study. The heterogeneity was moderate (I^2^ = 36.8%). The Q-value indicated no significant variability between studies (Q = 18.9, p = 0.09).Square size is proportional to study weight. Diamonds reflect the pooled effect sizes.

**Supplementary Figure 11. Meta-analysis unnested microglial density data, including newly generated data from the NBB and EBB cohort.** The forest plot shows all individual data points included in a random effects meta-analysis, representing effect sizes (Hedges’s g’) with 95 confidence interval (CI) for differences between SCZ patients and controls in each study. Square size is proportional to study weight. The column “Brain region” refers to the brain region studied (DLPFC-Dorsolateral Prefrontal Cortex; ACC-Anterior Cingulate Cortex; mACC-mid anterior Cingulate Cortex) and measurement (outcome given for each microglia staining marker used in each study).

**Supplementary Figure 12. Meta-analysis cellular density stratified per brain region, including newly generated data EBB and NBB cohort.** Forest plot of subgroup meta-analysis for microglia density in SCZ stratified per brain region (frontal cortex, regions related to the limbic system, occipital cortex, and temporal cortex), including the current analysis on samples from the NBB and EBB). The Q-value indicated significant variability between studies (Q = 10.84, p = 0.02).The column “marker” shows the marker that was used to assess microglial density in each study. Diamonds reflect the pooled effect sizes.

**Supplementary Figure 13. Meta-analysis microglia gene expression levels including newly generated data from the NBB and EBB cohorts.** The forest plot shows all individual data points included in a random effects meta-analysis, representing effect sizes (Hedges’s g’) with 95 confidence interval (CI) for differences between SCZ patients and controls in each study (including the current analysis on samples from the NBB and EBB). The heterogeneity was moderate to high (I^2^ = 54.8 %). The Q-value indicated significant variability between studies (Q = 64.2, p < 0.001). Square size is proportional to study weight. The column “marker” shows the marker that was used to assess microglial density in each study. Studies are ordered by marker. The column “Brain region” refers to the brain region studied (DLPFC-Dorsolateral Prefrontal Cortex; ACC-Anterior Cingulate Cortex; mACC-mid anterior Cingulate Cortex). Diamonds reflect the pooled effect sizes.

**Supplementary Figure 14. Meta-analysis microglia gene expression stratified per brain region, including newly generated data from EBB and NBB cohort.** Forest plot of subgroup meta-analysis for molecular markers in SCZ stratified per brain region (frontal cortex, regions related to the limbic system, and temporal cortex), including the current analysis on samples from the NBB and EBB. No differences between these groups were detected, as shown by the Q-test-based ANOVA (Q_between_ = 2.53, p = 0.28). The column “marker” shows the marker that was used to assess microglial density in each study. Diamonds reflect the pooled effect sizes.

**Supplementary Figure 15. Subgroup meta-analysis for cortical or subcortical brain regions, including additional data EBB and NBB cohort.** Forest plot of subgroup meta-analysis for molecular markers in SCZ stratified per global brain region (cortex versus subcortical). No differences between these groups were detected, as shown by the Q-test-based ANOVA (Q_between_ = 2.61, p = 0.106). The column “marker” shows gene expression level that has been measured in each study. Diamonds reflect the pooled effect sizes.

**Supplementary Figure 16. Subgroup meta-analysis for microglia gene expression stratified per marker including additional data EBB and NBB cohort.** Forest plots of subgroup meta-analysis on gene expression levels of microglial genes in schizophrenia (SCZ). The Q-value indicated significant variability between the marker studies (Q_between_ = 32.57, p = 0.008). The column “Brain region” refers to the specific brain region studied (DLPFC-Dorsolateral Prefrontal Cortex).

**Supplementary Figure 17. Flowchart.** Study selection for macrophage markers. These macrophage markers (*n* = 7) were selected because they have been more recently described as macrophage-specific markers (Bowman et al., 2016; Greter et al., 2015; Gu et al., 2016; Mrdjen et al., 2018).

**References:**

Bowman, R. L., Klemm, F., Akkari, L., Pyonteck, S. M., Sevenich, L., Quail, D. F., Dhara, S., Simpson, K., Gardner, E. E., Iacobuzio-Donahue, C. A., Brennan, C. W., Tabar, V., Gutin, P. H., & Joyce, J. A. (2016). Macrophage Ontogeny Underlies Differences in Tumor-Specific Education in Brain Malignancies. *Cell Reports*. https://doi.org/10.1016/j.celrep.2016.10.052

Busse, S., Busse, M., Schiltz, K., Bielau, H., Gos, T., Brisch, R., Mawrin, C., Schmitt, A., Jordan, W., Müller, U. J., Bernstein, H.-G., Bogerts, B., & Steiner, J. (2012). Different distribution patterns of lymphocytes and microglia in the hippocampus of patients with residual versus paranoid schizophrenia: further evidence for disease course-related immune alterations? *Brain, Behavior, and Immunity*, *26*(8), 1273–1279. https://doi.org/10.1016/j.bbi.2012.08.005

Durrenberger, P. F., Fernando, F. S., Kashefi, S. N., Bonnert, T. P., Seilhean, D., Nait-Oumesmar, B., Schmitt, A., Gebicke-Haerter, P. J., Falkai, P., Grünblatt, E., Palkovits, M., Arzberger, T., Kretzschmar, H., Dexter, D. T., & Reynolds, R. (2015). Common mechanisms in neurodegeneration and neuroinflammation: a BrainNet Europe gene expression microarray study. *Journal of Neural Transmission*. https://doi.org/10.1007/s00702-014-1293-0

Gos, T., Myint, A. M., Schiltz, K., Meyer-Lotz, G., Dobrowolny, H., Busse, S., Müller, U. J., Mawrin, C., Bernstein, H. G., Bogerts, B., & Steiner, J. (2014). Reduced microglial immunoreactivity for endogenous NMDA receptor agonist quinolinic acid in the hippocampus of schizophrenia patients. *Brain, Behavior, and Immunity*. https://doi.org/10.1016/j.bbi.2014.05.012

Greter, M., Lelios, I., & Croxford, A. L. (2015). Microglia versus myeloid cell nomenclature during brain inflammation. In *Frontiers in Immunology*. https://doi.org/10.3389/fimmu.2015.00249

Gu, N., Peng, J., Murugan, M., Wang, X., Eyo, U. B., Sun, D., Ren, Y., DiCicco-Bloom, E., Young, W., Dong, H., & Wu, L. J. (2016). Spinal Microgliosis Due to Resident Microglial Proliferation Is Required for Pain Hypersensitivity after Peripheral Nerve Injury. *Cell Reports*. https://doi.org/10.1016/j.celrep.2016.06.018

Mrdjen, D., Pavlovic, A., Hartmann, F. J., Schreiner, B., Utz, S. G., Leung, B. P., Lelios, I., Heppner, F. L., Kipnis, J., Merkler, D., Greter, M., & Becher, B. (2018). High-Dimensional Single-Cell Mapping of Central Nervous System Immune Cells Reveals Distinct Myeloid Subsets in Health, Aging, and Disease. *Immunity*. https://doi.org/10.1016/j.immuni.2018.01.011

Radewicz, K., Garey, L. J., Gentleman, S. M., & Reynolds, R. (2000). Increase in HLA-DR immunoreactive microglia in frontal and temporal cortex of chronic schizophrenics. *Journal of Neuropathology and Experimental Neurology*, *59*(2), 137–150.

Steiner, J., Bielau, H., Brisch, R., Danos, P., Ullrich, O., Mawrin, C., Bernstein, H.-G., & Bogerts, B. (2008). Immunological aspects in the neurobiology of suicide: Elevated microglial density in schizophrenia and depression is associated with suicide. *Journal of Psychiatric Research*, *42*(2), 151–157. https://doi.org/10.1016/j.jpsychires.2006.10.013

Steiner, J., Mawrin, C., Ziegeler, A., Bielau, H., Ullrich, O., Bernstein, H. G., & Bogerts, B. (2006). Distribution of HLA-DR-positive microglia in schizophrenia reflects impaired cerebral lateralization. *Acta Neuropathologica*. https://doi.org/10.1007/s00401-006-0090-8

Wierzba-Bobrowicz, T., Lewandowska, E., Lechowicz, W., Stepień, T., & Pasennik, E. (2005). Quantitative analysis of activated microglia, ramified and damage of processes in the frontal and temporal lobes of chronic schizophrenics. *Folia Neuropathologica*.
