# Supplementary material for: A loss of mature microglial markers without immune activation in schizophrenia
Source: Glia. 2021 Jan 7;69(5):1251–67. doi: 10.1002/glia.23962 (PMC7986895; doi:10.1002/glia.23962)
Supplement: Supplementary file 2 — Appendix S2. Figures. [file GLIA-69-1251-s001.docx]

# Figure S1.

EMBASE search (2/28/2020) N=204

PubMed search (2/28/2020) N=85

Duplicates removed

-

N=4

N=34

Cross referencing of inclusions

Excluded N=170

No human post-mortem brain tissue: 35 No schizophrenia patients: 29

No controls: 7

Screening of title and abstract N=204

No microglia density analysis: 33 Review/no original research: 64 Not in English: 1

No full-text available: 1

Screening full text N=38

N=15

Excluded N= 22

No schizophrenia patients: 1 No microglia density analysis: 2 Review/no original research: 2 Conference abstract: 15 Overlapping samples: 2*

Included in review N=16

Current study N =1


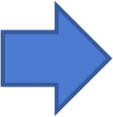


Included in quantitative analysis

N=12

Included in qualitative analysis

N=16

**Figure S2.**

**Microglial cell density (unnested)**

**Brain**

**region (specific)**

**Marker Study Statistics**

Hedges's Lower Upper

g limit limit p-Value

ACC HLA-DR Steiner et al. (2006) -0,076 -0,751 0,600 0,827

ACC HLA-DR Steiner et al. (2006) -0,299 -0,978 0,381 0,389

ACC HLA-DR Radewicz et al. (2000) 0,341 -0,454 1,136 0,400

ACC NOX2 Seredenina et al. (2017) 0,359 -0,355 1,073 0,325

ACC NOX2 Seredenina et al. (2017) -0,090 -0,799 0,618 0,803

Caudate nucleus CD68 Falke et al. (2000) -0,005 -0,793 0,784 0,990

DLPFC HLA-DR Fillman et al. (2013) 0,358 -0,127 0,842 0,148

DLPFC HLA-DR Steiner et al. (2006) 0,093 -0,583 0,768 0,788

DLPFC HLA-DR Steiner et al. (2006) -0,393 -1,075 0,289 0,259

DLPFC HLA-DR Radewicz et al. (2000) 0,990 0,152 1,829 0,021

DLPFC IBA-1 Hercher et al. (2014) 0,196 -0,413 0,805 0,527

DLPFC layer 3 HLA-DR Fillman et al. (2013) 0,030 -0,519 0,580 0,914

DLPFC layer 5/6 HLA-DR Fillman et al. (2013) 0,010 -0,441 0,461 0,967

Dorsal raphe nucleus HLA-DR Brisch et al. (2017) -0,228 -0,841 0,385 0,466

Entorhinal cortex CD68 Arnold et al. (1998) 0,186 -0,466 0,837 0,576

Frontal cortex (active) HLA-DR Wierzba-Bobrowicz et al. (2005) 1,516 0,403 2,630 0,008

Frontal cortex (ramified) HLA-DR Wierzba-Bobrowicz et al. (2005) 0,869 -0,151 1,890 0,095

Hippocampus HLA-DR Busse et al. (2012) 0,415 -0,330 1,159 0,275

Hippocampus HLA-DR Busse et al. (2012) 0,313 -0,428 1,054 0,407

Hippocampus CA1 CD68 Arnold et al. (1998) 0,039 -0,611 0,690 0,905

Hippocampus subiculum CD68 Arnold et al. (1998) 0,022 -0,628 0,672 0,948

mACC IBA-1 Petrasch-Parwez et al. (2020) -0,411 -1,075 0,253 0,225

Midfrontal cortex CD68 Arnold et al. (1998) -0,061 -0,712 0,589 0,853

Occipital cortex CD68 Arnold et al. (1998) -0,411 -1,068 0,245 0,220

Orbitofrontal cortex CD68 Arnold et al. (1998) 0,172 -0,479 0,823 0,604

Prefrontal cortex dorsal (activated macrophages) IBA-1 + CD68 Schnieder et al. (2014) -0,257 -1,010 0,497 0,504

Prefrontal cortex dorsal (resting microglia) IBA-1 + CD68 Schnieder et al. (2014) -0,155 -0,906 0,597 0,686

Prefrontal cortex ventral (activated macrophages) IBA-1 + CD68 Schnieder et al. (2014) -0,387 -1,144 0,371 0,317

Prefrontal cortex ventral (resting microglia) IBA-1 + CD68 Schnieder et al. (2014) 0,156 -0,595 0,908 0,684

Temporal cortex HLA-DR Schnieder et al. (2014) -0,696 -1,528 0,136 0,101

Temporal cortex HLA-DR Radewicz et al. (2000) 2,601 1,511 3,690 0,000

Temporal cortex (active) HLA-DR Wierzba-Bobrowicz et al. (2005) 2,365 1,076 3,654 0,000

Temporal cortex (ramified) HLA-DR Wierzba-Bobrowicz et al. (2005) 1,409 0,314 2,504 0,012

Thalamus (mediodorsal nucleus) CD68 Falke et al. (2000) 0,810 -0,012 1,633 0,054

**OVERALL**

**0,183 -0,000 0,365 0,050**

-5,00 -2,50 0,00 2,50 5,00

**Decrease Increase**

**Figure S3.**

**Microglial cell density (grouped by brain region and nested)**

**Brain region**

**Marker**

**Study**

**Statistics**

Hedges's Lower Upper

g limit limit p-Value

Frontal cortex Frontal cortex Frontal cortex Frontal cortex Frontal cortex Frontal cortex Frontal cortex

CD68 HLA-DR IBA-1 HLA-DR

IBA-1/CD68 HLA-DR HLA-DR

Arnold et al. (1998) 0,055 -0,595 0,706 0,868

Fillman et al. (2013) 0,133 -0,364 0,629 0,601


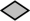

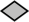


Hercher et al. (2014) 0,196 -0,413 0,805 0,527

Radewicz et al. (2000) 0,990 0,152 1,829 0,021

Schnieder et al. (2014) -0,161 -0,914 0,593 0,676

Steiner et al. (2006) -0,150 -0,829 0,529 0,665

Wierzba-Bobrowicz et al. (2005) 1,193 0,125 2,261 0,029

Frontal cortex 0,202 -0,062 0,465 0,133

Limbic system Limbic system Limbic system Limbic system Limbic system Limbic system Limbic system Limbic system

CD68 HLA-DR HLA-DR CD68 IBA-1 HLA-DR NOX2 HLA-DR

Arnold et al. (1998) 0,082 -0,568 0,733 0,804

Brisch et al. (2017) -0,228 -0,841 0,385 0,466

Busse et al. (2012) 0,364 -0,379 1,107 0,337

Falke et al. (2000) 0,403 -0,403 1,209 0,327

Petrasch-Parwez et al. (2020) -0,411 -1,075 0,253 0,225

Radewicz et al. (2000) 0,341 -0,454 1,136 0,400

Seredenina et al. (2017) 0,134 -0,577 0,845 0,712

Steiner et al. (2006) -0,187 -0,865 0,490 0,588

Limbic system 0,022 -0,231 0,276 0,864

Occipital cortex

CD68

Arnold et al. (1998) -0,411 -1,068 0,245 0,220

Occipital cortex -0,411 -1,087 0,264 0,233

Temporal cortex HLA-DR Temporal cortex HLA-DR

Radewicz et al. (2000) 2,601 1,511 3,690 0,000

Wierzba-Bobrowicz et al. (2005) 1,887 0,691 3,083 0,002

Temporal cortex 2,277 1,463 3,090 0,000

**OVERALL**

**0,436 -0,220 1,091 0,193**

-5,00 -2,50 0,00 2,50 5,00

**Decrease Increase**

**Figure S4.**

**Funnelplot of standard error by Hedges g’ (nested) in microglial cell density studies**

0,0

*

*

0,1

**Standard error**

0,2

0,3

0,4

0,5

0,6

-2,0 -1,5 -1,0 -0,5 0,0 0,5 1,0 1,5 2,0

**Hedges g’**

**Figure S5.**

PubMed search (2/28/2020) N=24

EMBASE search (2/28/2020) N=76

Duplicates removed

Excluded N=65

No human post-mortem brain tissue: 13 No schizophrenia patients: 8

No controls: 3

No microglia gene expression: 9 Review/no original research: 24 No full-text available: 5

Other: 3

Screening of title and abstract N=85

N=20

Cross-referencing of inclusions

N=2

Screening full-text N=22

N=7

Excluded N=15

No schizophrenia patients: 2

No microglia gene expression analysis: 11

Bias towards significant results: 2

Included in review N=7


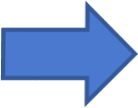


Current study N =1

Included in quantitative analysis N=7

Included in qualitative analysis N=7

**Figure S6.**

**Expression microglial markers (grouped by cortical or subcortical brain regions)**

**Brain region**

**Marker Study Statistics**

Hedges's Lower Upper

g limit limit p-Value

Cortex CD68 Lopez-Gonzalez et al. (2019) -0,845 -1,598 -0,093 0,028


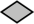

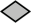

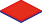


Cortex AIF1 Lopez-Gonzalez et al. (2019) -0,479 -1,209 0,251 0,198

Cortex CSF1R Lopez-Gonzalez et al. (2019) -0,874 -1,629 -0,119 0,023

Cortex TGFB1 Lopez-Gonzalez et al. (2019) -0,300 -1,023 0,424 0,417

Cortex NOX2 Seredenina et al. (2017) 0,021 -0,468 0,509 0,934

Cortex AIF1 Fiorentino et al. (2016) -1,324 -2,193 -0,456 0,003

Cortex HLA-DRA Durrenberger et al. (2015) -0,723 -1,659 0,213 0,130

Cortex HLA-DRB4 Durrenberger et al. (2015) -1,403 -2,522 -0,284 0,014

Cortex HLA-DRB4 Schmitt et al. (2011) -0,188 -1,235 0,860 0,726

Cortex HLA-DRA Nakatani et al. (2006) -0,555 -1,623 0,513 0,308

Cortex -0,553 -0,817 -0,290 0,000

Subcortical NOX2 Seredenina et al. (2017) -0,169 -0,657 0,320 0,499

Subcortical AIF1 Seredenina et al. (2017) -0,526 -1,015 -0,037 0,035

Subcortical CD68 Seredenina et al. (2017) -0,102 -0,590 0,386 0,683

Subcortical ITGAM Seredenina et al. (2017) -0,256 -0,735 0,223 0,295

Subcortical HLA-DRA Sinkus et al. (2013) -0,054 -0,467 0,358 0,797

Subcortical -0,212 -0,454 0,029 0,084

### OVERALL

#### -0,379 -0,712 -0,045 0,026

-5,00 -2,50 0,00 2,50 5,00

## Decrease Increase

**Figure S7.**

**Microglial gene expression (grouped by brain region)**

**Brain region**

**Marker Study Statistics**

Hedges's Lower Upper

g limit limit p-Value

Frontal cortex CD68 Lopez-Gonzalez et al. (2019) -0,845 -1,598 -0,093 0,028


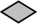

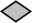

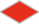


Frontal cortex AIF1 Lopez-Gonzalez et al. (2019) -0,479 -1,209 0,251 0,198

Frontal cortex CSF1R Lopez-Gonzalez et al. (2019) -0,874 -1,629 -0,119 0,023

Frontal cortex TGFB1 Lopez-Gonzalez et al. (2019) -0,300 -1,023 0,424 0,417

Frontal cortex NOX2 Seredenina et al. (2017) 0,021 -0,468 0,509 0,934

Frontal cortex AIF1 Fiorentino et al. (2016) -1,324 -2,193 -0,456 0,003

Frontal cortex HLA-DRA Nakatani et al. (2006) -0,555 -1,623 0,513 0,308

Frontal cortex -0,514 -0,810 -0,218 0,001

Limbic system NOX2 Seredenina et al. (2017) -0,169 -0,657 0,320 0,499

Limbic system AIF1 Seredenina et al. (2017) -0,526 -1,015 -0,037 0,035

Limbic system CD68 Seredenina et al. (2017) -0,102 -0,590 0,386 0,683

Limbic system ITGAM Seredenina et al. (2017) -0,256 -0,735 0,223 0,295

Limbic system HLA-DRA Sinkus et al. (2013) -0,054 -0,467 0,358 0,797

Limbic system -0,213 -0,461 0,035 0,092

Temporal cortex HLA-DRA Durrenberger et al. (2015) -0,723 -1,659 0,213 0,130

Temporal cortex HLA-DRB4 Durrenberger et al. (2015) -1,403 -2,522 -0,284 0,014

Temporal cortex HLA-DRB4 Schmitt et al. (2011) -0,188 -1,235 0,860 0,726

Temporal cortex -0,744 -1,361 -0,128 0,018

### OVERALL

#### -0,426 -0,740 -0,111 0,008

-5,00 -2,50 0,00 2,50 5,00

## Decrease Increase

**Figure S8.**

**Expression microglial gene expression (grouped by marker)**

**Marker Brain**

**region**

**Study**

**Statistics**

Hedges's Lower Upper

g limit limit p-Value

AIF1 AIF1 AIF1

DLPFC

Cingulate cortex Frontal cortex

Lopez-Gonzalez et al. (2019) -0,479 -1,209 0,251 0,198

Seredenina et al. (2017) -0,526 -1,015 -0,037 0,035


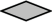

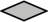


Fiorentino et al. (2016) -1,324 -2,193 -0,456 0,003

AIF1 -0,694 -1,168 -0,220 0,004

HLA-DR HLA-DR HLA-DR HLA-DR HLA-DR

Temporal cortex Temporal cortex Temporal cortex DLPFC

Hippocampus

Durrenberger et al. (2015) -0,723 -1,659 0,213 0,130

Durrenberger et al. (2015) -1,403 -2,522 -0,284 0,014

Schmitt et al. (2011) -0,188 -1,235 0,860 0,726

Nakatani et al. (2006) -0,555 -1,623 0,513 0,308

Sinkus et al. (2013) -0,054 -0,467 0,358 0,797

HLA-DR -0,411 -0,835 0,014 0,058

-5,00 -2,50 0,00 2,50 5,00

## Decrease Increase

**Figure S9.**

**Funnelplot of standard error by Hedges g’ microglial gene expression studies**

0,0

*

0,1

0,2

**Standard error**

0,3

0,4

0,5

0,6

-2,0 -1,5 -1,0 -0,5 0,0 0,5 1,0 1,5 2,0

## Hedges g’

**Figure S10.**

**Microglial cell density (nested, including current study)**

**Study Statistics**

Hedges's Lower Upper

g limit limit p-Value


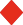


Current study 0,086 -0,641 0,813 0,817

Schnieder et al. (2014) -0,268 -1,037 0,502 0,496

Brisch et al. (2017) -0,228 -0,841 0,385 0,466

Seredenina et al. (2017) 0,134 -0,577 0,845 0,712

Hercher et al. (2014) 0,196 -0,413 0,805 0,527

Fillman et al. (2013) 0,133 -0,364 0,629 0,601

Busse et al. (2012) 0,364 -0,379 1,107 0,337

Steiner et al. (2006) -0,169 -0,847 0,510 0,626

Wierzba-Bobrowicz et al. (2005) 1,540 0,406 2,674 0,008

Falke et al. (2000) 0,403 -0,403 1,209 0,327

Radewicz et al. (2000) 1,311 0,394 2,228 0,005

Arnold et al. (1998) -0,009 -0,661 0,643 0,979

Petrasch-Parwez et al. (2020) -0,411 -1,075 0,253 0,225

**OVERALL**

**0,144 -0,102 0,390 0,250**

-5,00 -2,50 0,00 2,50 5,00

**Decrease Increase**

**Figure S11.**

**Microglial cell density (unnested, including current study)**

**Brain region**

**Marker Study Statistics**

Hedges's Lower Upper

g limit limit p-Value

ACC NOX2 Seredenina et al. (2017) 0,359 -0,355 1,073 0,325


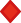


ACC NOX2 Seredenina et al. (2017) -0,090 -0,799 0,618 0,803

ACC HLA-DR Steiner et al. (2006) -0,076 -0,751 0,600 0,827

ACC HLA-DR Steiner et al. (2006) -0,299 -0,978 0,381 0,389

ACC HLA-DR Radewicz et al. (2000) 0,341 -0,454 1,136 0,400

Caudate nucleus CD68 Falke et al. (2000) -0,005 -0,793 0,784 0,990

DLPFC IBA-1 Hercher et al. (2014) 0,196 -0,413 0,805 0,527

DLPFC HLA-DR Fillman et al. (2013) 0,358 -0,127 0,842 0,148

DLPFC HLA-DR Steiner et al. (2006) 0,093 -0,583 0,768 0,788

DLPFC HLA-DR Steiner et al. (2006) -0,393 -1,075 0,289 0,259

DLPFC HLA-DR Radewicz et al. (2000) 0,990 0,152 1,829 0,021

DLPFC layer 3 HLA-DR Fillman et al. (2013) 0,030 -0,519 0,580 0,914

DLPFC layer 5/6 HLA-DR Fillman et al. (2013) 0,010 -0,441 0,461 0,967

Dorsal raphe nucleus HLA-DR Brisch et al. (2017) -0,228 -0,841 0,385 0,466

Entorhinal cortex CD68 Arnold et al. (1998) 0,186 -0,466 0,837 0,576

Frontal cortex (active) HLA-DR Wierzba-Bobrowicz et al. (2005) 1,516 0,403 2,630 0,008

Frontal cortex (ramified) HLA-DR Wierzba-Bobrowicz et al. (2005) 0,869 -0,151 1,890 0,095

Hippocampus HLA-DR Busse et al. (2012) 0,415 -0,330 1,159 0,275

Hippocampus HLA-DR Busse et al. (2012) 0,313 -0,428 1,054 0,407

Hippocampus CA1 CD68 Arnold et al. (1998) 0,039 -0,611 0,690 0,905

Hippocampus subiculum CD68 Arnold et al. (1998) 0,022 -0,628 0,672 0,948

mACC IBA-1 Petrasch-Parwez et al. (2020) -0,411 -1,075 0,253 0,225

Midfrontal cortex CD68 Arnold et al. (1998) -0,061 -0,712 0,589 0,853

Occipital cortex CD68 Arnold et al. (1998) -0,411 -1,068 0,245 0,220

Orbitofrontal cortex CD68 Arnold et al. (1998) 0,172 -0,479 0,823 0,604

Prefrontal cortex dorsal (activated macrophages) IBA-1 + CD68 Schnieder et al. (2014) -0,257 -1,010 0,497 0,504

Prefrontal cortex dorsal (resting microglia) IBA-1 + CD68 Schnieder et al. (2014) -0,155 -0,906 0,597 0,686

Prefrontal cortex ventral (activated macrophages) IBA-1 + CD68 Schnieder et al. (2014) -0,387 -1,144 0,371 0,317

Prefrontal cortex ventral (resting microglia) IBA-1 + CD68 Schnieder et al. (2014) 0,156 -0,595 0,908 0,684

Temporal cortex IBA-1 Current study 0,086 -0,641 0,813 0,817

Temporal cortex HLA-DR Schnieder et al. (2014) -0,696 -1,528 0,136 0,101

Temporal cortex HLA-DR Radewicz et al. (2000) 2,601 1,511 3,690 0,000

Temporal cortex (active) HLA-DR Wierzba-Bobrowicz et al. (2005) 2,365 1,076 3,654 0,000

Temporal cortex (ramified) HLA-DR Wierzba-Bobrowicz et al. (2005) 1,409 0,314 2,504 0,012

Thalamus (mediodorsal nucleus) CD68 Falke et al. (2000) 0,810 -0,012 1,633 0,054

**OVERALL**

**0,126 0,007 0,245 0,037**

-5,00 -2,50 0,00 2,50 5,00

**Decrease Increase**

**Figure S12.**

**Microglial cell density grouped by brain region (nested, including current study)**

**Brain region**

**Marker Study Statistics**

Hedges's Lower Upper

g limit limit p-Value

Frontal cortex Frontal cortex Frontal cortex Frontal cortex Frontal cortex Frontal cortex Frontal cortex

CD68 HLA-DR IBA-1 HLA-DR

IBA-1/CD68 HLA-DR HLA-DR

Arnold et al. (1998) 0,055 -0,595 0,706 0,868

Fillman et al. (2013) 0,133 -0,364 0,629 0,601


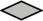

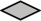


Hercher et al. (2014) 0,196 -0,413 0,805 0,527

Radewicz et al. (2000) 0,990 0,152 1,829 0,021

Schnieder et al. (2014) -0,161 -0,914 0,593 0,676

Steiner et al. (2006) -0,150 -0,829 0,529 0,665

Wierzba-Bobrowicz et al. (2005) 1,193 0,125 2,261 0,029

Frontal cortex 0,246 -0,138 0,630 0,210

Limbic system Limbic system Limbic system Limbic system Limbic system Limbic system Limbic system Limbic system

CD68 HLA-DR HLA-DR CD68 IBA-1 HLA-DR NOX2 HLA-DR

Arnold et al. (1998) 0,082 -0,568 0,733 0,804

Brisch et al. (2017) -0,228 -0,841 0,385 0,466

Busse et al. (2012) 0,364 -0,379 1,107 0,337

Falke et al. (2000) 0,403 -0,403 1,209 0,327

Petrasch-Parwez et al. (2020) -0,411 -1,075 0,253 0,225

Radewicz et al. (2000) 0,341 -0,454 1,136 0,400

Seredenina et al. (2017) 0,134 -0,577 0,845 0,712

Steiner et al. (2006) -0,187 -0,865 0,490 0,588

Limbic system 0,041 -0,317 0,399 0,821

Occipital cortex

CD68

Arnold et al. (1998) -0,411 -1,068 0,245 0,220

Occipital cortex -0,411 -1,391 0,569 0,411

Temporal cortex Temporal cortex Temporal cortex

IBA-1 HLA-DR HLA-DR

Current study 0,086 -0,641 0,813 0,817

Radewicz et al. (2000) 2,601 1,511 3,690 0,000

Wierzba-Bobrowicz et al. (2005) 1,887 0,691 3,083 0,002

Temporal cortex 1,256 0,555 1,956 0,000

**OVERALL**

**0,300 -0,255 0,856 0,289**

-5,00 -2,50 0,00 2,50 5,00

**Decrease Increase**

**Figure S13.**

**Microglial marker expression (unnested, including current study)**

**Marker Brain Study Statistics region**

**Hedges'sLower Upper**

**g limit limit p-Value**


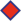


AIF1 DLPFC Lopez-Gonzalez et al. (2019) -0,479 -1,209 0,251 0,198

AIF1 Cingulate cortex Seredenina et al. (2017) -0,526 -1,015 -0,037 0,035

CD68 Temporal cortex Current study -0,458 -1,276 0,360 0,272

CD68 DLPFC Lopez-Gonzalez et al. (2019) -0,845 -1,598 -0,093 0,028

CD68 Cingulate cortex Seredenina et al. (2017) -0,102 -0,590 0,386 0,683

CSF1R Temporal cortex Current study -1,141 -2,013 -0,269 0,010

CSF1R DLPFC Lopez-Gonzalez et al. (2019) -0,874 -1,629 -0,119 0,023

CX3CR1 Temporal cortex Current study -0,658 -1,487 0,171 0,120

GPR34 Temporal cortex Current study 0,233 -0,577 1,043 0,573

HLA-DRA Temporal cortex Durrenberger et al. (2015) -0,723 -1,659 0,213 0,130

HLA-DRA DLPFC Nakatani et al. (2006) -0,555 -1,623 0,513 0,308

HLA-DRA Hippocampus Sinkus et al. (2013) -0,054 -0,467 0,358 0,797

HLA-DRB4 Temporal cortex Durrenberger et al. (2015) -1,403 -2,522 -0,284 0,014

HLA-DRB4 Temporal cortex Schmitt et al. (2011) -0,188 -1,235 0,860 0,726

IBA-1 Temporal cortex Current study 0,353 -0,460 1,167 0,395

IRF8 Temporal cortex Current study -1,202 -2,081 -0,324 0,007

ITGAM Temporal cortex Current study -0,726 -1,559 0,108 0,088

ITGAM Cingulate cortex Seredenina et al. (2017) -0,256 -0,735 0,223 0,295

ITGAX Temporal cortex Current study -0,883 -1,729 -0,036 0,041

ITGB2 Temporal cortex Current study -1,015 -1,873 -0,156 0,021

NOX2 Prefrontal cortex Seredenina et al. (2017) 0,021 -0,468 0,509 0,934

NOX2 Cingulate cortex Seredenina et al. (2017) -0,169 -0,657 0,320 0,499

OLR1 Temporal cortex Current study -1,550 -2,473 -0,627 0,001

P2YR12 Temporal cortex Current study -1,893 -2,868 -0,918 0,000

TGFB1 Temporal cortex Current study 0,289 -0,523 1,100 0,485

TGFB1 DLPFC Lopez-Gonzalez et al. (2019) -0,300 -1,023 0,424 0,417

TMEM119 Temporal cortex Current study -1,256 -2,141 -0,371 0,005

TREM2 Temporal cortex Current study -0,097 -0,905 0,711 0,814

TYROBP Temporal cortex Current study 0,731 -0,104 1,565 0,086

**OVERALL**

**-0,478 -0,678 -0,279 0,000**

-5,00 -2,50 0,00 2,50 5,00

**Decrease Increase**

**Figure S14.**

**Expression microglial gene expression grouped by brain region (unnested, including current study)**

**Brain region**

**Marker Study Statistics**

Hedges's Lower Upper

g limit limit p-Value

Frontal cortex CD68 Lopez-Gonzalez et al. (2019) -0,845 -1,598 -0,093 0,028


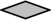

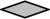

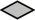

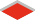


Frontal cortex AIF1 Lopez-Gonzalez et al. (2019) -0,479 -1,209 0,251 0,198

Frontal cortex CSF1R Lopez-Gonzalez et al. (2019) -0,874 -1,629 -0,119 0,023

Frontal cortex TGFB1 Lopez-Gonzalez et al. (2019) -0,300 -1,023 0,424 0,417

Frontal cortex NOX2 Seredenina et al. (2017) 0,021 -0,468 0,509 0,934

Frontal cortex AIF1 Fiorentino et al. (2016) -1,324 -2,193 -0,456 0,003

Frontal cortex HLA-DRA Nakatani et al. (2006) -0,555 -1,623 0,513 0,308

Frontal cortex -0,573 -0,981 -0,165 0,006

Limbic system NOX2 Seredenina et al. (2017) -0,169 -0,657 0,320 0,499

Limbic system AIF1 Seredenina et al. (2017) -0,526 -1,015 -0,037 0,035

Limbic system CD68 Seredenina et al. (2017) -0,102 -0,590 0,386 0,683

Limbic system ITGAM Seredenina et al. (2017) -0,256 -0,735 0,223 0,295

Limbic system HLA-DRA Sinkus et al. (2013) -0,054 -0,467 0,358 0,797

Limbic system -0,218 -0,623 0,186 0,290

Temporal cortex CX3CR1 Current study -0,658 -1,487 0,171 0,120

Temporal cortex CD68 Current study -0,458 -1,276 0,360 0,272

Temporal cortex P2YR12 Current study -1,893 -2,868 -0,918 0,000

Temporal cortex ITGAM Current study -0,726 -1,559 0,108 0,088

Temporal cortex ITGB2 Current study -1,015 -1,873 -0,156 0,021

Temporal cortex ITGAX Current study -0,883 -1,729 -0,036 0,041

Temporal cortex TMEM119 Current study -1,256 -2,141 -0,371 0,005

Temporal cortex GPR34 Current study 0,233 -0,577 1,043 0,573

Temporal cortex CSF1R Current study -1,141 -2,013 -0,269 0,010

Temporal cortex IRF8 Current study -1,202 -2,081 -0,324 0,007

Temporal cortex TGFB1 Current study 0,289 -0,523 1,100 0,485

Temporal cortex IBA-1 Current study 0,353 -0,460 1,167 0,395

Temporal cortex TREM2 Current study -0,097 -0,905 0,711 0,814

Temporal cortex OLR1 Current study -1,550 -2,473 -0,627 0,001

Temporal cortex TYROBP Current study 0,731 -0,104 1,565 0,086

Temporal cortex HLA-DRA Durrenberger et al. (2015) -0,723 -1,659 0,213 0,130

Temporal cortex HLA-DRB4 Durrenberger et al. (2015) -1,403 -2,522 -0,284 0,014

Temporal cortex HLA-DRB4 Schmitt et al. (2011) -0,188 -1,235 0,860 0,726

Temporal cortex -0,606 -0,881 -0,330 0,000

**OVERALL**

#### -0,485 -0,760 -0,211 0,001

-5,00 -2,50 0,00 2,50 5,00

## Decrease Increase

**Figure S15.**

**Expression microglial markers (including current study, grouped by cortical and subcortical brain regions)**

**Brain region**

**Marker**

**Study**

**Statistics**

#### Hedges's Lower Upper

**g limit limit p-Value**

Cortex CX3CR1 Current study -0,658 -1,487 0,171 0,120


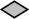

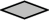

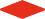


Cortex CD68 Current study -0,458 -1,276 0,360 0,272

Cortex P2YR12 Current study -1,893 -2,868 -0,918 0,000

Cortex ITGAM Current study -0,726 -1,559 0,108 0,088

Cortex ITGB2 Current study -1,015 -1,873 -0,156 0,021

Cortex ITGAX Current study -0,883 -1,729 -0,036 0,041

Cortex TMEM119 Current study -1,256 -2,141 -0,371 0,005

Cortex GPR34 Current study 0,233 -0,577 1,043 0,573

Cortex CSF1R Current study -1,141 -2,013 -0,269 0,010

Cortex IRF8 Current study -1,202 -2,081 -0,324 0,007

Cortex TGFB1 Current study 0,289 -0,523 1,100 0,485

Cortex IBA-1 Current study 0,353 -0,460 1,167 0,395

Cortex TREM2 Current study -0,097 -0,905 0,711 0,814

Cortex OLR1 Current study -1,550 -2,473 -0,627 0,001

Cortex TYROBP Current study 0,731 -0,104 1,565 0,086

Cortex CD68 Lopez-Gonzalez et al. (2019) -0,845 -1,598 -0,093 0,028

Cortex AIF1 Lopez-Gonzalez et al. (2019) -0,479 -1,209 0,251 0,198

Cortex CSF1R Lopez-Gonzalez et al. (2019) -0,874 -1,629 -0,119 0,023

Cortex TGFB1 Lopez-Gonzalez et al. (2019) -0,300 -1,023 0,424 0,417

Cortex NOX2 Seredenina et al. (2017) 0,021 -0,468 0,509 0,934

Cortex AIF1 Fiorentino et al. (2016) -1,324 -2,193 -0,456 0,003

Cortex HLA-DRA Durrenberger et al. (2015) -0,723 -1,659 0,213 0,130

Cortex HLA-DRB4 Durrenberger et al. (2015) -1,403 -2,522 -0,284 0,014

Cortex HLA-DRB4 Schmitt et al. (2011) -0,188 -1,235 0,860 0,726

Cortex HLA-DRA Nakatani et al. (2006) -0,555 -1,623 0,513 0,308

Cortex -0,594 -0,819 -0,369 0,000

Subcortical NOX2 Seredenina et al. (2017) -0,169 -0,657 0,320 0,499

Subcortical AIF1 Seredenina et al. (2017) -0,526 -1,015 -0,037 0,035

Subcortical CD68 Seredenina et al. (2017) -0,102 -0,590 0,386 0,683

Subcortical ITGAM Seredenina et al. (2017) -0,256 -0,735 0,223 0,295

Subcortical HLA-DRA Sinkus et al. (2013) -0,054 -0,467 0,358 0,797

Subcortical -0,218 -0,614 0,177 0,280

**OVERALL**

#### -0,443 -0,804 -0,082 0,016

-5,00 -2,50 0,00 2,50 5,00

## Decrease Increase

**Figure S16.**

**Expression microglial markers (including current study, grouped by marker)**

**Marker Brain region Study Statistics**

**Hedges's Lower Upper**

**g limit limit p-Value**


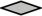

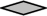

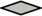


AIF1 Temporal cortex Current study 0,353 -0,460 1,167 0,395

AIF1 DLPFC Lopez-Gonzalez et al. (2019) -0,479 -1,209 0,251 0,198

AIF1 Cingulate cortex Seredenina et al. (2017) -0,526 -1,015 -0,037 0,035

AIF1 Frontal cortex Fiorentino et al. (2016) -1,324 -2,193 -0,456 0,003

AIF1 -0,484 -0,910 -0,058 0,026

CD68 Temporal cortex Current study -0,458 -1,276 0,360 0,272

CD68 DLPFC Lopez-Gonzalez et al. (2019) -0,845 -1,598 -0,093 0,028

CD68 Cingulate cortex Seredenina et al. (2017) -0,102 -0,590 0,386 0,683

CD68 -0,398 -0,872 0,076 0,100

HLA-DR Temporal cortex Durrenberger et al. (2015) -0,723 -1,659 0,213 0,130

HLA-DR Temporal cortex Durrenberger et al. (2015) -1,403 -2,522 -0,284 0,014

HLA-DR Temporal cortex Schmitt et al. (2011) -0,188 -1,235 0,860 0,726

HLA-DR DLPFC Nakatani et al. (2006) -0,555 -1,623 0,513 0,308

HLA-DR Hippocampus Sinkus et al. (2013) -0,054 -0,467 0,358 0,797

HLA-DR -0,413 -0,839 0,014 0,058

-5,00 -2,50 0,00 2,50 5,00

**Decrease Increase**

# Figure S17.

**Search strategy:**

**(**postmortem OR post-mortem OR autopsy) AND (CCR2 OR ITGA4 OR CD49d OR CD44 OR CD169 OR CD38 OR CD11a OR PDL1 OR SIGLEC1 OR ITGAL OR CD274)

AND (schizophreni* OR psychotic OR psychosis)

PubMed search (13/10/2020) N=0

EMBASE search (13/10/2020) N=0

Screening of title and abstract N=0
