# Supplementary material for: A loss of mature microglial markers without immune activation in schizophrenia
Source: Glia. 2021 Jan 7;69(5):1251–67. doi: 10.1002/glia.23962 (PMC7986895; doi:10.1002/glia.23962)
Supplement: Supplementary file 3 — Appendix S3. Tables. [file GLIA-69-1251-s003.docx]

**Supplementary Table 1. Summary of the demographics of the postmortem brain donors of the Netherlands Brain Bank and the Edinburgh Brain Bank**. Information is separated by experiment. None of the variables were significantly different between the controls and SCZ group although the p-value was 0.06 for age in RNA expression cohort.

|  |  | **Control (N=16)** | **SCZ (N=12)** | **p-value** |
| --- | --- | --- | --- | --- |
| **Immunohistochemistry DAB staining** | Age (years) | 70.6 (13.0) | 69.8 (13.6) | 0.875 |
|  | Sex (F:M) | 6:10 | 6:6 | 0.508 |
|  | PMI (hours) | 24.2 (30.9) | 16.3 (15.6) | 0.426 |
|  | pH | 6.5 (0.33) | 6.7 (0.34) | 0.129 |
|  |  | **Control (N=19)** | **SCZ (N=18)** |  |
| **IBA1/TMEM119 staining** | Age (years) | 68.8 (14.0) | 66.6 (13.7) | 0.632 |
|  | Sex (F:M) | 13:6 | 12:6 | 0.909 |
|  | PMI (hours) | 21.8 (29.3) | 26.1 (25.3) | 0.636 |
|  | pH | 6.7 (0.34) | 6.6 (0.81) | 0.624 |
|  |  | **Control (N=14)** | **SCZ (N=9)** |  |
| **mRNA expression** | Age (years) | 73.1 ± 17.0 | 60.8 ± 10.4 | 0.066 |
|  | Sex (M:F) | 8:6 | 4:5 | 0.552 |
|  | PMD (hours) | 28.5 ± 34.7 | 21.2 ± 23.4 | 0.586 |
|  | pH | 6.60 ± 0.30 | 6.34 ± 0.58 | 0.171 |
| PMI = post-mortem interval. Numbers represent mean ± standard deviation. M = males, F = females, SCZ = schizophrenia. P-value is based on an independent t-test or Chi-Square test. | | | |  |

**Supplementary Table 2. Detailed clinicopathological overview of the post-mortem brain donors of the Netherlands Brain Bank and the Edinburgh Brain Bank.**

| Status | Donor | Sex | Age | PMD (hours) | pH | Cause of death* | Experiment |
| --- | --- | --- | --- | --- | --- | --- | --- |
| Control | 00-067 | M | 73 | 25 | NA | 2 | 1, 2 |
| Control | 01-158 | F | 69 | 6 | 6.97 | 4 | 1,2 |
| Control | 04-019 | M | 91 | 39 | 6.61 | 4 | 1,2 |
| Control | 04-081 | M | 67 | 19 | 6.70 | 2 | 1,2 |
| Control | 05-019 | M | 74 | 5 | 6.70 | 2 | 1,2 |
| Control | 05-073 | F | 87 | 6 | 6.96 | 7 | 1 |
| Control | 09-003 | M | 62 | 7 | 6.36 | 7 | 1,2 |
| Control | 09-300 | F | 71 | 70 | 6.31 | 9 | 1,3 |
| Control | 11-069 | M | 49 | 6 | 6.23 | 4 | 1,2 |
| Control | 11-096 | F | 70 | 6 | 6.55 | 3 | 1,2 |
| Control | 12-052 | F | 64 | 5 | 6.35 | 2 | 1,2 |
| Control | 12-104 | M | 79 | 6 | 6.71 | 4 | 1,2 |
| Control | 13-056 | M | 95 | 7 | 6.56 | 2 | 1,2 |
| Control | 99-144 | F | 59 | 4 | 6.67 | 6 | 1,2 |
| Control | 96-238 | F | 87 | 8 | 6.91 | 2 | 3 |
| Control | 96-251 | M | 84 | 9 | 6.2 | 2 | 3 |
| Control | 03-009 | M | 51 | 8 | NA | NA | 3 |
| Control | 09-039 | M | 78 | 18 | 6.52 | 2 | 3 |
| Control | 09-301 | M | 92 | 8 | 6.14 | 2 | 3 |
| Control | 11-028 | F | 81 | 4 | 6.67 | 9 | 3 |
| Control | 11-039 | F | 91 | 4 | 6.5 | 2 | 3 |
| Control | 11-044 | M | 51 | 8 | 7.05 | 5 | 2,3 |
| Control | 12-001 | F | 89 | 6 | 6.75 | 8a | 3 |
| Control | 12-005 | F | 84 | 6 | 6.68 | 8a | 3 |
| Control | SD015-12 | M | 70 | 73 | 6.90 | 2 | 1,2,3 |
| Control | SD019-15 | M | 50 | 103 | 5.70 | 2 | 1,2,3 |
| Control | SD022-13 | M | 45 | 74 | 6.60 | 2 | 2,3 |
| Control | 05-273 | F | 87 | 6 | 6.96 | NA | 2 |
| Control | 96-129 | M | 70 | 7.5 | 6.4 | 3 | 2 |
| Control | 14-029 | F | 78 | 7 | 6.32 | 4 | 2 |
| SCZ | 05-161 | F | 66 | 11 | 7.24 | 3 | 1,2,3 |
| SCZ | 09-286 | M | 78 | 3 | NA | 2,4e | 1,2 |
| SCZ | 10-049 | M | 59 | 13 | 5.93 | 2 | 2,3 |
| SCZ | 10-158 | M | 64 | 19 | 7 | 2 | 1,2,3 |
| SCZ | 10-360 | F | 79 | 5 | 6.34 | 2 | 1,2,3 |
| SCZ | 12-031 | F | 55 | 9 | 6.82 | 4 | 1,2,3 |
| SCZ | 13-006 | F | 63 | 5 | 6.50 | 3 | 1,2,3 |
| SCZ | 93-274 | F | 68 | 10 | 6.45 | 1,6 | 1,2,3 |
| SCZ | 98-127 | F | 85 | 9 | NA | 2 | 1,2 |
| SCZ | 99-182 | M | 67 | 17 | NA | 2 | 1,2 |
| SCZ | 99-209 | M | 74 | 52 | NA | 1 | 1,2 |
| SCZ | 99-262 | M | 95 | 12 | NA | 2 | 1,2 |
| SCZ | SD017-06 | M | 44 | 44 | 6.40 | 2 | 1,2,3 |
| SCZ | SD035-08 | M | 50 | 75 | 6.1 | 2 | 2,3 |
| SCZ | 98-229 | M | 76 | 87 | NA | 2 | 2 |
| SCZ | 98-329 | M | 48 | 41 | NA | 9 | 2 |
| SCZ | SD033-07 | M | 50 | 45 | 6.28 | 2 | 2 |
| SCZ | 94-098 | F | 66 | 11 | 7.24 | 3 | 2 |
| Control = control donor; SCZ = donor with schizophrenia; M = male; F = female; NA = not applicable; - = absent; PMD = post-mortem delay; cause of death: 1 = infection/inflammatory; 2 = cardiorespiratory; 3 = cancer; 4 = euthanasia/palliative sedation*; 5 = suicide;. 6 trauma; 7. Dehydration; 8 Brain a) ischemia/infarction, b) bleeding; 9 = other (ileus, organ failure). Experiment: 1. Iba1 staining; 2. Iba1/TMEM119 staining; 3. qPCR. * euthanasia is legal according to Dutch law | | | | | | | |

**Supplementary Table 3. qRT-PCR Primers used in this study.**

| Gene | Forward | Reverse | Description |
| --- | --- | --- | --- |
| *AIF1* | AGACGTTCAGCTACCCTGACTT | GGCCTGTTGGCTTTTCCTTTTCTC | Microglia/myeloid cell marker |
| *CD68* | CTTCTCTCATTCCCCTATGGACA | GAAGGACACATTGTACTCCACC | Microglia/myeloid cell marker |
| *CSF1R* | ATCAGCATCCGGCTGAAAGT | CTCGAATCCGCACCAGCTCT | Microglia/myeloid cell marker |
| *CX3CR1* | CTTACGATGGCACCCAGTGA | CAAGGCAGTCCAGGAGAGTT | Microglia/myeloid cell marker |
| *GPR34* | CCTGATGTCCAGTAACATTCGC | CATGCAGGGAGTATCCTGGT | Microglia/myeloid cell marker |
| *HLA-DRA* | CCCAGGGAAGACCACCTTT | CACCCTGCAGTCGTAAACGT | Microglia/myeloid cell marker |
| *IFR8* | ATCAAAAGGAGCCCTTCCCC | TAGGTGGTGTACCCCGTCAC | Microglia/myeloid cell marker |
| *ITGAM* | TGCTTCCTGTTTGGATCCAACCTA | AGAAGGCAATGTCACTATCCTCTTGA | Microglia/myeloid cell marker |
| *ITGAX* | GCTCCCGGTGAAGTATGCTG | CCACATGGCTTTCCTTCTCCT | Microglia/myeloid cell marker |
| *ITGB2* | CCTCAACGAGATCACCGAGT | CAGCTTATCAGGGTGCGTGT | Microglia/myeloid cell marker |
| *OLR1* | AGTTCGTGACTGCTTCACTCTCTC | CTTCTCATCAGGCTGGTCCTT | Microglia/myeloid cell marker |
| *P2RY12* | TTTGTGTGTCAAGTTACCTCCG | CTGGTGGTCTTCTGGTAGCG | Microglia cell marker |
| *TGFB1* | CAATTCCTGGCGATACCTCAG | GCACAACTCCGGTGACATCAA | Microglia/myeloid cell marker |
| *TMEM119* | CTTCCTGGATGGGATAGTGGAC | GCACAGACGATGAACATCAGC 9 | Microglia marker |
| *TREM2* | TCAGGAAGGTCCTGGTGGA | GGGTGGGAAGGGGATTTCTC | Microglia/myeloid cell marker |
| *TYROBP* | TACGGCCTCTGTGTGTTGAG | CGGAAACAGCGTATCACTGAG | Microglia/myeloid cell marker |
| *ACTB* | GTGGACATCCGCAAAGACCT | TCTGCATCCTGTCGGCAAT | Housekeeping gene |
| *GAPDH* | TGCACCACCAACTGCTTAGC | GGCATGGACTGTGGTCATGA | Housekeeping gene |
| *SDHA* | GAAGCCCTTTGAGGAGCACT | GTTTTGTCGATCACGGGTCT | Housekeeping gene |

**Supplementary Table 4. Inclusion and exclusion full-text screening microglia cell density.**

| Title | Authors | Details | Possible inclusion | No human postmortem brain tissue | No schizophrenia patients | No controls | No microglial density analysis | Review/no original research | Not in English | | Conference Abstract |
| --- | --- | --- | --- | --- | --- | --- | --- | --- | --- | --- | --- |
| Decreased NOX2 expression in the brain of patients with bipolar disorder: association with valproic acid prescription and substance abuse. | Seredenina T, Sorce S, Herrmann FR, Ma Mulone XJ, Plastre O, Aguzzi A, Jaquet V, Krause KH. | Transl Psychiatry. 2017 Aug 15 | 1 |  |  |  |  |  |  |  | |
| Microglia in the dorsal raphe nucleus plays a potential role in both suicide facilitation and prevention in affective disorders. | Brisch R, Steiner J, Mawrin C, KrzyÅ¼anowska M, Jankowski Z, Gos T. | Eur Arch Psychiatry Clin Neurosci. 2017 Aug | 1 |  |  |  |  |  |  |  | |
| Evidence for morphological alterations in prefrontal white matter glia in schizophrenia and bipolar disorder. | Hercher C, Chopra V, Beasley CL. | J Psychiatry Neurosci. 2014 Nov | 1 |  |  |  |  |  |  |  | |
| Reduced microglial immunoreactivity for endogenous NMDA receptor agonist quinolinic acid in the hippocampus of schizophrenia patients. | Gos T, Myint AM, Schiltz K, Meyer-Lotz G, Dobrowolny H, Busse S, MÃ¼ller UJ, Mawrin C, Bernstein HG, Bogerts B, Steiner J. | Brain Behav Immun. 2014 Oct | 1^a^ |  |  |  |  |  |  |  | |
| Different distribution patterns of lymphocytes and microglia in the hippocampus of patients with residual versus paranoid schizophrenia: further evidence for disease course-related immune alterations? | Busse S, Busse M, Schiltz K, Bielau H, Gos T, Brisch R, Mawrin C, Schmitt A, Jordan W, MÃ¼ller UJ, Bernstein HG, Bogerts B, Steiner J. | Brain Behav Immun. 2012 Nov | 1^a^ |  |  |  |  |  |  |  | |
| Calprotectin in microglia from frontal cortex is up-regulated in schizophrenia: evidence for an inflammatory process? | Foster R, Kandanearatchi A, Beasley C, Williams B, Khan N, Fagerhol MK, Everall IP. | Eur J Neurosci. 2006 Dec | 1 |  |  |  |  |  |  |  | |
| Immunological aspects in the neurobiology of suicide: elevated microglial density in schizophrenia and depression is associated with suicide. | Steiner J, Bielau H, Brisch R, Danos P, Ullrich O, Mawrin C, Bernstein HG, Bogerts B. | J Psychiatr Res. 2008 Jan | 1^b^ |  |  |  |  |  |  |  | |
| Distribution of HLA-DR-positive microglia in schizophrenia reflects impaired cerebral lateralization. | Steiner J, Mawrin C, Ziegeler A, Bielau H, Ullrich O, Bernstein HG, Bogerts B. | Acta Neuropathol. 2006 Sep | 1^b^ |  |  |  |  |  |  |  | |
| Increase in HLA-DR immunoreactive microglia in frontal and temporal cortex of chronic schizophrenics. | Radewicz K, Garey LJ, Gentleman SM, Reynolds R. | J Neuropathol Exp Neurol. 2000 Feb | 1 |  |  |  |  |  |  |  | |
| Absence of neurodegeneration in the thalamus and caudate of elderly patients with schizophrenia. | Falke E, Han LY, Arnold SE. | Psychiatry Res. 2000 Mar 6 | 1 |  |  |  |  |  |  |  | |
| Evidence for activation of microglia in patients with psychiatric illnesses. | Bayer TA, Buslei R, Havas L, Falkai P. | Neurosci Lett. 1999 Aug 20 | 1 |  |  |  |  |  |  |  | |
| Absence of neurodegeneration and neural injury in the cerebral cortex in a sample of elderly patients with schizophrenia. | Arnold SE, Trojanowski JQ, Gur RE, Blackwell P, Han LY, Choi C. | Arch Gen Psychiatry. 1998 Mar | 1 |  |  |  |  |  |  |  | |
| The hypothalamus and neuropsychiatric disorders: psychiatry meets microscopy | Bernstein H.-G., Dobrowolny H., Bogerts B., Keilhoff G., Steiner J. | Cell and Tissue Research (2019) 375:1 (243-258). Date of Publication: 28 Jan 2019 |  |  |  |  |  | 1 |  |  | |
| Active HHV-6 infection of cerebellar Purkinje cells in mood disorders | Prusty B.K., Gulve N., Govind S., Krueger G.R.F., Feichtinger J., Larcombe L., Aspinall R., Ablashi D.V., Toro C.T. | Frontiers in Microbiology (2018) 9:AUG Article Number: 1955. Date of Publication: 21 Aug 2018 |  |  |  |  | 1 |  |  |  | |
| ICAM-1 is increased in brain and peripheral levels of soluble ICAM-1 is related to cognitive deficits in schizophrenia | Weickert T., Cai H., O'Donnell M., Balzan R., Wells R., Liu D., Galletly C., Weickert C.S. | Schizophrenia Bulletin (2018) 44 Supplement 1 (S73-S74). Date of Publication: 1 Apr 2018 |  |  |  |  |  |  |  | 1 | |
| Examination of optically cleared postmortem human brain samples for 3D analysis of molecular networks | Chang E., Yeung L.W., Birnbaum A., Malhotra A. | Neuropsychopharmacology (2017) 43 Supplement 1 (S593). Date of Publication: 1 Nov 2017 |  |  |  |  |  |  |  | 1 | |
| Microglia in post-mortem brain tissue of schizophrenia: Normal at first, but different when taking a more closer look | Sneeboer M., Akkerman N., Van Der Geest A., Litjens M., MacIntyre D., Palmen S.J.M.C., Kahn R., Hol E.M., De Witte L. | GLIA (2017) 65 Supplement 1 (E425-E426). Date of Publication: 1 Jun 2017 |  |  |  |  |  |  |  | 1 | |
| Revisiting microglia activation in psychiatric illnesses: A postmortem approach | Tonelli L.H., Soroka J., Vaughn C., Nicholson J.D. | Biological Psychiatry (2015) 77:9 SUPPL. 1 (91S-92S). Date of Publication: 1 May 2015 |  |  |  |  |  |  |  | 1 | |
| Autopsy studies of frontal white matter in schizophrenia | Dwork A.J., Hikita-Dwork C., John Mann J., Rosoklija G., Schnieder T., Stankov A., Trencevska-Ivanovska I., Zhang Z. | Schizophrenia Bulletin (2015) 41 SUPPL. 1 (S286). Date of Publication: March 2015 |  |  |  |  |  |  |  | 1 | |
| Multi-immunostaining for microglial activation in schizophrenia | De Picker L., Dumont G., Morrens M., Sabbe B.G.C., Gentleman S.M., Nicoll J.A.R., Boche D. | European Neuropsychopharmacology (2014) 24 SUPPL. 2 (S188-S189). Date of Publication: October 2014 |  |  |  |  |  |  |  | 1 | |
| Microglial activation in white matter in schizophrenia: Findings from a postmortem electron microscopic morphometric study | Uranova N.A., Vikhreva O.V., Rachmanova V.I., Orlovskaya D.D. | Neurology Psychiatry and Brain Research (2014) 20:1 (25). Date of Publication: February 2014 |  |  |  |  |  |  |  | 1 | |
| Immunohistochemical investigations of microglia in schizophrenia | Schmitt A., Bayer T., Falkai P. | European Archives of Psychiatry and Clinical Neuroscience (2013) 263:1 SUPPL. 1 (S21). Date of Publication: September 2013 |  |  |  |  |  |  |  | 1 | |
| HLA-DR + microglia are increased in paranoid schizophrenia versus residual schizophrenia: Evidence from human postmortem studies | Steiner J., Busse S., Bernstein H.-G., Bogerts B. | European Archives of Psychiatry and Clinical Neuroscience (2013) 263:1 SUPPL. 1 (S21). Date of Publication: September 2013 |  |  |  |  |  |  |  | 1 | |
| Ultrastructural alterations of myelinated fibers and oligodendrocytes in schizophrenia | Uranova N., Shklover V., Vikhreva O., Rachmanova V., Orlovskaya D., Kazanskiy P., Chelpanov V. | European Archives of Psychiatry and Clinical Neuroscience (2013) 263:1 SUPPL. 1 (S42). Date of Publication: September 2013 |  |  |  |  |  |  |  | 1 | |
| Microglial cell populations in prefrontal grey and white matter in schizophrenia and bipolar disorder | Beasley C., Hercher C. | Biological Psychiatry (2013) 73:9 SUPPL. 1 (263S). Date of Publication: 1 May 2013 |  |  |  |  |  |  |  | 1 | |
| White matter neuron alterations in schizophrenia and related disorders | Connor C.M., Crawford B.C., Akbarian S. | International Journal of Developmental Neuroscience (2011) 29:3 (325-334). Date of Publication: May 2011 |  |  |  |  |  | 1 |  |  | |
| The role of microglial cells in schizophrenia-current knowledge and future therapeutic approaches | Steiner J. | Schizophrenia Bulletin (2011) 37 SUPPL. 1 (186). Date of Publication: March 2011 |  |  |  |  |  |  |  | 1 | |
| Astrocyte expression of D2-like dopamine receptors in the prefrontal cortex | Mladinov M., Mayer D., Brcic L., Wolstencroft E., Man N.T., Holt I., Hof P.R., Morris G.E., Šimic G. | Translational Neuroscience (2010) 1:3 (238-243). Date of Publication: September 2010 |  |  | 1 |  |  |  |  |  | |
| Significance of glia cell dysfunction in schizophrenia | Bogerts B., Steiner J., Bernstein H.G. | European Archives of Psychiatry and Clinical Neuroscience (2010) 260 SUPPL. 1 (S24). Date of Publication: June 2010 |  |  |  |  |  |  |  | 1 | |
| Widespread microglia activation in recent onset schizophrenia | Van Berckel B., Yaqub M., Schulte A., Bossong M., Boellaard R., Kloet R., Schuitemaker A., Van Haren N., Luurtsema G., Windhorst B., Cahn W., Lammertsma A., Rozemuller A., Kahn R. | Journal of Cerebral Blood Flow and Metabolism (2009) 29 SUPPL. 1 (S590). Date of Publication: October 2009 |  |  |  |  |  |  |  | 1 | |
| Cingulate White Matter Neurons in Schizophrenia and Bipolar Disorder | Connor C.M., Guo Y., Akbarian S. | Biological Psychiatry (2009) 66:5 (486-493). Date of Publication: 1 Sep 2009 | 1 |  |  |  |  |  |  |  | |
| Microglia is widespread activated in recent onset schizophrenia: Evidence from PET and post mortem studies | Van Berckel B., Yaqub M., Bossong M., Boellaard R., Schuitemaker A., Lammertsma A.A., Windhorst A.D., Rozemuller A., Cahn W., Kahn R.S. | European Neuropsychopharmacology (2009) 19 SUPPL. 3 (S585). Date of Publication: 2009 |  |  |  |  |  |  |  | 1 | |
| Lateralization of increased density of Iba1-immunopositive microglial cells in the anterior midcingulate cortex of schizophrenia and bipolar disorder. | Petrasch-Parwez E, Schöbel A, Benali A, Moinfar Z, Förster E, Brüne M, Juckel G. | Eur Arch Psychiatry Clin Neurosci. 2020 Feb 15. doi: 10.1007/s00406-020-01107-0. [Epub ahead of print] | 1 |  |  |  |  |  |  |  | |
| [Microglial activation in schizophrenia: Is translocator 18 kDa protein (TSPO) the right marker?](https://www.ncbi.nlm.nih.gov/pubmed/31699629) | Sneeboer MAM, van der Doef T, Litjens M, Psy NBB, Melief J, Hol EM, Kahn RS, de Witte LD. | Schizophr Res. 2020 Jan;215:167-172. doi: 10.1016/j.schres.2019.10.045. Epub 2019 Nov 4. |  |  |  |  | 1 |  |  |  | |
| Increased inflammatory markers identified in the dorsolateral prefrontal cortex of individuals with schizophrenia. | Fillman SG, [Cloonan N](https://www.ncbi.nlm.nih.gov/pubmed/?term=Cloonan%20N%5BAuthor%5D&cauthor=true&cauthor_uid=22869038), [Catts VS](https://www.ncbi.nlm.nih.gov/pubmed/?term=Catts%20VS%5BAuthor%5D&cauthor=true&cauthor_uid=22869038), [Miller LC](https://www.ncbi.nlm.nih.gov/pubmed/?term=Miller%20LC%5BAuthor%5D&cauthor=true&cauthor_uid=22869038), [Wong J](https://www.ncbi.nlm.nih.gov/pubmed/?term=Wong%20J%5BAuthor%5D&cauthor=true&cauthor_uid=22869038), [McCrossin T](https://www.ncbi.nlm.nih.gov/pubmed/?term=McCrossin%20T%5BAuthor%5D&cauthor=true&cauthor_uid=22869038), [Cairns M](https://www.ncbi.nlm.nih.gov/pubmed/?term=Cairns%20M%5BAuthor%5D&cauthor=true&cauthor_uid=22869038), [Weickert CS](https://www.ncbi.nlm.nih.gov/pubmed/?term=Weickert%20CS%5BAuthor%5D&cauthor=true&cauthor_uid=22869038). | Mol Psychiatry (2013) | 1 |  |  |  |  |  |  |  | |
| Quantitative analysis of activated microglia, ramified and damage of processes in the frontal and temporal lobes of chronic schizophrenics. | Wierzba-Bobrowicz T, [Lewandowska E](https://www.ncbi.nlm.nih.gov/pubmed/?term=Lewandowska%20E%5BAuthor%5D&cauthor=true&cauthor_uid=16012909), [Lechowicz W](https://www.ncbi.nlm.nih.gov/pubmed/?term=Lechowicz%20W%5BAuthor%5D&cauthor=true&cauthor_uid=16012909), [Stepień T](https://www.ncbi.nlm.nih.gov/pubmed/?term=Stepie%C5%84%20T%5BAuthor%5D&cauthor=true&cauthor_uid=16012909), [Pasennik E](https://www.ncbi.nlm.nih.gov/pubmed/?term=Pasennik%20E%5BAuthor%5D&cauthor=true&cauthor_uid=16012909). | Folia Neuropathol. (2005) | 1 |  |  |  |  |  |  |  | |
| Regional differences in human ependymal and subventricular zone cytoarchitecture are unchanged in neuropsychiatric disease. | Comte I, [Kotagiri P](https://www.ncbi.nlm.nih.gov/pubmed/?term=Kotagiri%20P%5BAuthor%5D&cauthor=true&cauthor_uid=22831917), Szele FG. | Dev Neurosci. (2012) | 1 |  |  |  |  |  |  |  | |
| Microglia of Prefrontal White Matter in Suicide | Schnieder TP,  [Trencevska](https://www.ncbi.nlm.nih.gov/pubmed/?term=Trencevska%20I%5BAuthor%5D&cauthor=true&cauthor_uid=25101704) I,  [Rosoklija](https://www.ncbi.nlm.nih.gov/pubmed/?term=Rosoklija%20G%5BAuthor%5D&cauthor=true&cauthor_uid=25101704) G, [Stankov](https://www.ncbi.nlm.nih.gov/pubmed/?term=Stankov%20A%5BAuthor%5D&cauthor=true&cauthor_uid=25101704) A., Mann JJ, [Smiley](https://www.ncbi.nlm.nih.gov/pubmed/?term=Smiley%20J%5BAuthor%5D&cauthor=true&cauthor_uid=25101704) J, Dwork AJ. | J Neuropathol Exp Neurol. (2014) | 1 |  |  |  |  |  |  |  | |
| ^a^Overlap of samples between Gos et al., 2014 and Busse et al., 2012. Included study with largest sample size. ^b^Overlap of samples between Steiner et al., 2006 and Steiner et al., 2008. Included study with largest sample size. | | | | | | | | | | | |

**Supplementary Table 5. Qualitative assessment studies included meta-analysis cell density**

| First author | Year | Neuropathology^1^ | General confounders^2^ | Correction for age, gender, PMI^3^ | Extra confounders^4^ | Description methods^5^ | Diagnosis schizophrenia^6^ | Description demographics^7^ | Reporting Outcome^8^ |
| --- | --- | --- | --- | --- | --- | --- | --- | --- | --- |
| Arnold | 1998 | + | + | c | + | + | + | + | +/- |
| Bayer | 1999 | - | - | - | - | - | +/- | +/- | +/- |
| Radewicz | 2000 | + | + | c | - | - | + | + | +/- |
| Falke | 2000 | + | + | m,c | - | - | + | + | +/- |
| Wierzba-Bobrowicz | 2005 | - | - | - | + | + | +/- | - | + |
| Foster | 2006 | - | + | m | + | + | +/- | +/- | +/- |
| Steiner | 2006 | + | + | m,c | + | + | + | +/- | + |
| Connor | 2009 | + | - | m | + | + | + | - | +/- |
| Comte | 2012 | + | + | m | + | + | + | +/- | +/- |
| Busse | 2012 | + | + | m,c | + | + | + | +/- | + |
| Fillman | 2013 | + | + | m | + | + | + | +/- | + |
| Hercher | 2014 | + | + | c | + | + | + | +/- | +/- |
| Schnieder | 2014 | + | + | c | + | + | + | +/- | +/- |
| Brisch | 2017 | + | + | c | + | + | + | +/- | + |
| Seredenina | 2017 | + | + | m,c | + | + | + | +/- | +/- |
| Petrasch-Parwez | 2020 | + | + | m,c | + | + | + | +/- | +/- |
| *Description of qualitative categories:*  ^1^ Neuropathology: + donors checked for neuropathology and report thereof; - donors not checked for neuropathology or no report thereof.  ^2^ General confounders: + Reporting on age, sex and post-mortem interval (PMI) for SCZ and control groups; +/- 1 or 2 of these variables missing; - no information  ^3^ Matching/correction for confounders applied: m = matching applied; c = analysis of the impact of confounders and applied correction if necessary; - did not check for influence age and PMI  ^4^ Extra confounders: + additional confounders reported, such as medication use, pH, cause of death, ilness duration, age of onset; - no information  ^5^ Description methods: + Complete description of technical methodology and analyses (for replication purposes); +/- missing information; - no information  ^6^ Diagnosis schizophrenia: + Prospective based on structured interview or psychiatric assessment; +/- Retrospective based on charts; - No information reported  ^7^ Description demographics: + individual demographics given; +/- only provided on group level; - no information  ^8^ Reporting outcomes: + quantitative data reported; +/- value extraction from graphs required; - no values available for comparison microglia density SCZ vs control | | | | | | | | | |

**Supplementary Table 6. Studies included in meta-analysis cell density**

| First author | Year | Included in  Van Kesteren? | Brain bank* | N control | N SCZ | Age controls  (mean years) | Age SCZ  (mean years) | Sex control (male\|female) | Sex SCZ (male\|female) | PMI control  (mean hours) | PMD SCZ  (mean hours) | pH control | pH SCZ |
| --- | --- | --- | --- | --- | --- | --- | --- | --- | --- | --- | --- | --- | --- |
| Arnold | 1998 | Yes | PEN | 14 | 23 | 75.3 | 79.8 | 6\|8 | 8\|15 | 11.40 | 11.00 | NR | NR |
| Falke | 2000 | Yes | PEN | 11 | 12 | 77.6 | 80.5 | 7\|4 | 3\|9 | 12.40 | 10.70 | NR | NR |
| Radewicz | 2000 | Yes | CCH and Wiesloch | 10 | 8 | 72 | 80 | NR | NR | NR | NR | NR | NR |
| Wierzba-Bobrowicz | 2005 | Yes | MAG | 6 | 9 | 56.3 | 55.7 | NR | 0\|9 | NR | NR | NR | NR |
| Steiner | 2006 | Yes | MAG | 16 | 16 | 57.7 | 54.5 | 8\|8 | 8\|8 | 36.60 | 43.10 | NR | NR |
| Busse | 2012 | Yes | MAG | 11 | 17 | 56 | 52 | 6\|5 | 9\|8 | 39.00 | 44.00 | NR | NR |
| Fillman | 2013 | Yes | NWS | 37 | 29 | 51.1 | 51.3 | 30\|7 | 24\|13 | 24.80 | 28.46 | 6.66 | 6.61 |
| Hercher | 2014 | Yes | SMRI | 20 | 20 | 45.3 | 44.7 | 14\|6 | 13\|7 | 29.90 | 30.70 | 6.70 | 6.50 |
| Schnieder | 2014 | No | MAC | 18 | 10 | 47.6 | 61.8 | 4\|6 | 12\|6 | 15.30 | 13.10 | 6.50 | 6.25 |
| Brisch | 2017 | No | MAG | 22 | 18 | 52.6 | 50.5 | 7\|15 | 11\|7 | 33.95 | 38.28 | NR | NR |
| Seredenina | 2017 | No | SMRI | 14 | 15 | 48.1 | 44.2 | 9\|6 | 9\|6 | 23.70 | 33.70 | 6.30 | 6.10 |
| Petrasch-Parwez | 2020 | No | SMRI | 17 | 17 | 45.4 | 45.3 | 13\|4 | 11\| 6 | 29.1 | 30.40 | 6.69 | 6.49 |
| * PEN = University of Pensylvania; CCH = Charing Cross Hospital, London; Wiesloch = The State Psychiatric Hospital, Wiesloch, Germany; MAG = Magdeburg Brain Collection; NSW = New South Wales Tissue Resource Center; SMRI = Stanley Medical Research Institute’s brain collection; MAC = Macedonian/New York State Psychiatric Institute Brain Collection. NR = not reported. | | | | | | | | | | | | | |

**Supplementary Table 7. Quantitative data extracted from studies included in meta-analysis cell density**

| First author | Year | N control | N SCZ | Marker used | Counting method | Density measure | Brain region | Density  Mean control | Density  STDEV control | Density  Mean SCZ | Density  STDEV SCZ | Significant differences |
| --- | --- | --- | --- | --- | --- | --- | --- | --- | --- | --- | --- | --- |
| Arnold | 1998 | 14 | 23 | CD68 | Automated | cells/0.1 mm3 | Ento Cortex | 7.0 | 4.9 | 8.0 | 5.9 | N |
| Arnold | 1998 | 14 | 23 | CD68 | Automated | cells/0.1 mm3 | HIPP (SUB) | 11.3 | 8.9 | 11.5 | 9.1 | N |
| Arnold | 1998 | 14 | 23 | CD68 | Automated | cells/0.1 mm3 | HIPP (CA1) | 11.5 | 8.6 | 11.8 | 9.1 | N |
| Arnold | 1998 | 14 | 23 | CD68 | Automated | cells/0.1 mm3 | MFC | 5.2 | 3.3 | 5.0 | 4.3 | N |
| Arnold | 1998 | 14 | 23 | CD68 | Automated | cells/0.1 mm3 | OFC | 5.0 | 2.9 | 5.7 | 4.6 | N |
| Arnold | 1998 | 14 | 23 | CD68 | Automated | cells/0.1 mm3 | Occ Cortex | 3.4 | 2.1 | 2.6 | 1.8 | N |
| Falke | 2000 | 11 | 12 | CD68 | Automated | cells/mm2 | THA (MD) | 50.3 | 41.8 | 80.8 | 30.4 | N |
| Falke | 2000 | 11 | 12 | CD68 | Automated | cells/mm2 | Caudate Nuc | 44.7 | 16.8 | 44.6 | 22.5 | N |
| Radewicz | 2000 | 10 | 8 | HLA-DR | Manual | cells/mm2 | DLPFC | 89.0 | 15.8 | 115.0 | 25.5 | Y |
| Radewicz | 2000 | 10 | 8 | HLA-DR | Manual | cells/mm2 | Temp Cortex | 88.0 | 15.8 | 139.0 | 17.0 | Y |
| Radewicz | 2000 | 10 | 8 | HLA-DR | Manual | cells/mm2 | ACC | 129.6 | 45.0 | 144.3 | 38.3 | N |
| Wierzba-Bobrowicz | 2005 | 6 | 9 | HLA-DP/DQ/DR | Not mentioned | cells/mm2 | Front.cortex (ramified) | 3.3 | 3.4 | 10.1 | 9.0 | N |
| Wierzba-Bobrowicz | 2005 | 6 | 9 | HLA-DP/DQ/DR | Not mentioned | cells/mm2 | Front cortex (activated) | 5.6 | 4.5 | 20.7 | 11.4 | Y |
| Wierzba-Bobrowicz | 2005 | 6 | 9 | HLA-DP/DQ/DR | Not mentioned | cells/mm2 | Temp. cortex (ramified) | 11.8 | 10.9 | 34.0 | 16.8 | Y |
| Wierzba-Bobrowicz | 2005 | 6 | 9 | HLA-DP/DQ/DR | Not mentioned | cells/mm2 | Temp cortex (activated) | 13.3 | 7.8 | 46.6 | 15.7 | Y |
| Steiner | 2006 | 16 | 16 | HLA-DR | Manual | cells/mm2 | DLPFC (left) | 11.3 | 11.6 | 12.5 | 13.9 | N |
| Steiner | 2006 | 16 | 16 | HLA-DR | Manual | cells/mm2 | DLPFC (right) | 16.8 | 20.3 | 10.5 | 8.1 | N |
| Steiner | 2006 | 16 | 16 | HLA-DR | Manual | cells/mm2 | ACC (left) | 22.4 | 27.9 | 20.0 | 34.8 | N |
| Steiner | 2006 | 16 | 16 | HLA-DR | Manual | cells/mm2 | ACC (right) | 29.5 | 38.0 | 19.2 | 28.8 | N |
| Busse | 2012 | 11 | 17 | HLA-DR | Manual | cells/mm3 | HIPP (left) | 13.2 | 15.1 | 23.0 | 26.7 | N |
| Busse | 2012 | 11 | 17 | HLA-DR | Manual | cells/mm3 | HIPP (right) | 16.3 | 15.3 | 22.2 | 20.2 | N |
| Fillman | 2013 | 37 | 29 | HLA-DP/DQ/DR | Manual | cells/mm2 | DLPFC-adjacent white matter | 102.9 | 23.9 | 111.4 | 28.1 | N |
| Fillman | 2013 | 28 | 22 | HLA-DP/DQ/DR | Manual | cells/mm2 | DLPFC (layer 3) | 47.9 | 19.9 | 48.5 | 20.6 | N |
| Fillman | 2013 | 28 | 22 | HLA-DP/DQ/DR | Manual | cells/mm2 | DLPFC layer 5/6 | 50.0 | 21.6 | 50.2 | 22.1 | N |
| Hercher | 2014 | 20 | 20 | IBA1 | Manual | cells/mm2 | DLPFC-adjacent white matter | 126.6 | 26.6 | 132.6 | 33.1 | N |
| Schnieder | 2014 | 18 | 10 | IBA1 and CD68 | Automated | cells/mm3 | PFC (white, dorsal, activated) | 6657.2 | 2684.5 | 5895.7 | 3215.6 | N |
| Schnieder | 2014 | 18 | 10 | IBA1 and CD68 | Automated | cells/mm3 | PFC (white, dorsal, rest. Microglia) | 6385.5 | 2632.1 | 5973.0 | 2498.0 | N |
| Schnieder | 2014 | 18 | 10 | IBA1 and CD68 | Automated | cells/mm3 | PFC (white, ventral, activated) | 6540.6 | 2856.7 | 5414.5 | 2769.5 | N |
| Schnieder | 2014 | 18 | 10 | IBA1 and CD68 | Automated | cells/mm3 | PFC (white, ventral, resting) | 6744.1 | 2470.9 | 7137.0 | 2381.9 | N |
| Brisch | 2017 | 22 | 18 | HLA-DR | Manual | cells/mm2 | DNR | 393.9 | 526.1 | 295.9 | 233.6 | N |
| Seredenina | 2017 | 14 | 15 | NOX2 | Automated | cells/mm2 | ACC (grey) | 179.4 | 90.2 | 238.1 | 203.2 | N |
| Seredenina | 2017 | 14 | 15 | NOX2 | Automated | cells/mm2 | ACC (white) | 1219.2 | 471.5 | 1155.0 | 845.9 | N |
| Petrasch-Parwez | 2020 | 17 | 17 | IBA1 | Manual | cells/mm3 | MCC | 6020.0 | 1229.0 | 5529.0 | 1100.0 | N |
| Ento Cortex = Entorhinal cortex ; HIPP = hippocampus; SUB = subiculum; MFC = midfrontal cortex; OFC = orbitofrontal cortex; Occ cortex = occipital cortex; Caud Nucleus = Caudate nucleus; THA = thalamus; MD = Mediodorsal nucleus; DLPFC = dorsolateral prefrontal cortex; Temp cortex = temporal cortex; PFC = prefrontal cortex; ACC = anterior cingulate cortex; MCC = midcingulate cortex; DNR = Dorsal raphe nucleus. Significant difference in microglia density per brain region between SCZ patients and controls based on standardized difference in means: N = no, Y = yes. | | | | | | | | | | | |  |

**Supplementary Table 8. Overview of studies reporting on morphology**

| Author | Year | N control | N SCZ | Region(s) | Brain Bank | Markers used | Description morphology |
| --- | --- | --- | --- | --- | --- | --- | --- |
| Radewicz | 2000 | 10 | 8 | DLPFC | CCH and Wiesloch | HLA-DR | In contrast to microglial cells in Alzheimer’s disease, the morphology of microglia in schizophrenia is not drastically altered. A tendency towards an increased number of processes was noted. |
| Wierzba-Bobrowicz | 2005 | 6 | 9 | Front and temp cortex | NR | HLA-DP/DQ/DR | Increased densities of both ramified and activated microglial cells were found in schizophrenia |
| Hercher | 2014 | 20 | 20 | DLPFC-adjacent white matter | SMRI | Iba1 | A qualitative assessment of microglial morphology found numerous activated microglial cells in 3 schizophrenia samples, but not in control samples. (χ2= 0.198, p = 0.656) |
| Schnieder | 2014 | 18 | 10 | Prefrontal cortex | MAC | Iba1/CD68 | In all areas analyzed, densities of activated cells correlated positively with total cell density. The percentage of activated microglia cells in the ventral and dorsal prefrontal white matter was not significantly different between SCZ and controls (dorsal: 48% versus 50%, p=0.737, ventral: 41% versus 48%, p=0.362) |
| Brisch | 2017 | 22 | 18 | Dorsal raphne nucleus | MAG | HLA-DR | Different morphological forms were observed, but not specifically related to any of the analysed groups or subgroups of patients. |
| Petrasch-Parwez | 2020 | 17 | 17 | ACC | SMRI | Iba1 | Iba1+ cells displayed mainly a ramified, primed (both resting) and reactive phenotype, while amoeboid cells or microglial nodules were rarely observed. Differences in the activation state were detected, but were also observed in individuals of three groups (BD, SCZ, controls) without any obvious attribution neither to one of the three groups nor to the left or right aMCC. |
| * CCH = Charing Cross Hospital, London; Wiesloch = The State Psychiatric Hospital, Wiesloch, Germany; MAG = Magdeburg Brain Collection; SMRI = Stanley Medical Research Institute’s brain collection; MAC = Macedonian/New York State Psychiatric Institute Brain Collection. | | | | | | | |

| Grey matter | SCZ patients  (n = 9) | Controls  (n = 16) | p-value* |
| --- | --- | --- | --- |
| Ramified and primed, n (%) | 6 (66.6%) | 12 (75%) | 0.194 |
| Reactive and amoeboid, n (%) | 3 (33.3%) | 4 (25%) |  |
| *p-value based on chi-square test. Morphological assessment is extensively described in (Torres-Platas et al., 2014)  *Torres-Platas SG, Cruceanu C, Chen GG, Turecki G, Mechawar N. (2014). Evidence for increased microglial priming and macrophage recruitment in the dorsal anterior cingulate white matter of depressed suicides. Brain Behav Immun 42:50–59.* | | | |

**Supplementary Table 9. Morphological assessment in grey matter of superior temporal gyrus of schizophrenia patients and controls**

**Supplementary Table 10. Inclusion and exclusion full-text screening microglial gene expression analysis**.

| Title | Description | Details | Inclusion | No human postmortem brain tissue | No schizophrenia patients | No controls | No microglia gene expression | Review/no original research/conference abstract | Not in English | Bias towards significant results |
| --- | --- | --- | --- | --- | --- | --- | --- | --- | --- | --- |
| Blood-brain barrier and intestinal epithelial barrier alterations in autism spectrum disorders. | Fiorentino M, Sapone A, Senger S, Camhi SS, Kadzielski SM, Buie TM, Kelly DL, Cascella N, Fasano A. | Mol Autism. 2016 Nov 29;7:49. eCollection 2016. | 1 |  |  |  |  |  |  |  |
| Cingulate white matter neurons in schizophrenia and bipolar disorder. | Connor CM, Guo Y, Akbarian S. | Biol Psychiatry. 2009 Sep 1;66(5):486-93. doi: 10.1016/j.biopsych.2009.04.032. Epub 2009 Jun 25. |  |  |  |  | 1 |  |  |  |
| Decreased NOX2 expression in the brain of patients with bipolar disorder: association with valproic acid prescription and substance abuse. | Seredenina T, Sorce S, Herrmann FR, Ma Mulone XJ, Plastre O, Aguzzi A, Jaquet V, Krause KH. | Transl Psychiatry. 2017 Aug 15;7(8):e1206. doi: 10.1038/tp.2017.175. | 1 |  |  |  |  |  |  |  |
| Different distribution patterns of lymphocytes and microglia in the hippocampus of patients with residual versus paranoid schizophrenia: further evidence for disease course-related immune alterations? | Busse S, Busse M, Schiltz K, Bielau H, Gos T, Brisch R, Mawrin C, Schmitt A, Jordan W, MÃ¼ller UJ, Bernstein HG, Bogerts B, Steiner J. | Brain Behav Immun. 2012 Nov;26(8):1273-9. doi: 10.1016/j.bbi.2012.08.005. Epub 2012 Aug 14. |  |  |  |  | 1 |  |  |  |
| Differential expression of synaptic and interneuron genes in the aging human prefrontal cortex. | Mohan A, Thalamuthu A, Mather KA, Zhang Y, Catts VS, Weickert CS, Sachdev PS. | Neurobiol Aging. 2018 Oct;70:194-202. doi: 10.1016/j.neurobiolaging.2018.06.011. Epub 2018 Jul 19. |  |  | 1 |  |  |  |  |  |
| Distribution of HLA-DR-positive microglia in schizophrenia reflects impaired cerebral lateralization. | Steiner J, Mawrin C, Ziegeler A, Bielau H, Ullrich O, Bernstein HG, Bogerts B. | Acta Neuropathol. 2006 Sep;112(3):305-16. Epub 2006 Jun 17. |  |  |  |  | 1 |  |  |  |
| Evidence for activation of microglia in patients with psychiatric illnesses. | Bayer TA, Buslei R, Havas L, Falkai P. | Neurosci Lett. 1999 Aug 20;271(2):126-8. |  |  |  |  | 1 |  |  |  |
| Evidence for morphological alterations in prefrontal white matter glia in schizophrenia and bipolar disorder. | Hercher C, Chopra V, Beasley CL. | J Psychiatry Neurosci. 2014 Nov;39(6):376-85. |  |  |  |  | 1 |  |  |  |
| Genome-wide expression analysis detects eight genes with robust alterations specific to bipolar I disorder: relevance to neuronal network perturbation. | Nakatani N, Hattori E, Ohnishi T, Dean B, Iwayama Y, Matsumoto I, Kato T, Osumi N, Higuchi T, Niwa S, Yoshikawa T. | Hum Mol Genet. 2006 Jun 15;15(12):1949-62. Epub 2006 May 10. | 1 |  |  |  |  |  |  |  |
| Immunological aspects in the neurobiology of suicide: elevated microglial density in schizophrenia and depression is associated with suicide. | Steiner J, Bielau H, Brisch R, Danos P, Ullrich O, Mawrin C, Bernstein HG, Bogerts B. | J Psychiatr Res. 2008 Jan;42(2):151-7. Epub 2006 Dec 15. |  |  |  |  | 1 |  |  |  |
| Increase in HLA-DR immunoreactive microglia in frontal and temporal cortex of chronic schizophrenics. | Radewicz K, Garey LJ, Gentleman SM, Reynolds R. | J Neuropathol Exp Neurol. 2000 Feb;59(2):137-50. |  |  |  |  | 1 |  |  |  |
| Innate immune response is differentially dysregulated between bipolar disease and schizophrenia. | de Baumont A, Maschietto M, Lima L, Carraro DM, Olivieri EH, Fiorini A, Barreta LA, Palha JA, Belmonte-de-Abreu P, Moreira Filho CA, Brentani H. | Schizophr Res. 2015 Feb;161(2-3):215-21. doi: 10.1016/j.schres.2014.10.055. Epub 2014 Dec 6. |  |  |  |  |  |  |  | 1 |
| Microglia in the dorsal raphe nucleus plays a potential role in both suicide facilitation and prevention in affective disorders. | Brisch R, Steiner J, Mawrin C, KrzyÅ¼anowska M, Jankowski Z, Gos T. | Eur Arch Psychiatry Clin Neurosci. 2017 Aug;267(5):403-415. doi: 10.1007/s00406-017-0774-1. Epub 2017 Feb 22. |  |  |  |  | 1 |  |  |  |
| Neuroinflammation in the dorsolateral prefrontal cortex in elderly chronic schizophrenia. | LÃ³pez-GonzÃ¡lez I, Pinacho R, Vila Ãˆ, Escanilla A, Ferrer I, Ramos B. | Eur Neuropsychopharmacol. 2019 Mar;29(3):384-396. doi: 10.1016/j.euroneuro.2018.12.011. Epub 2019 Jan 7. | 1 |  |  |  |  |  |  |  |
| Reduced microglial immunoreactivity for endogenous NMDA receptor agonist quinolinic acid in the hippocampus of schizophrenia patients. | Gos T, Myint AM, Schiltz K, Meyer-Lotz G, Dobrowolny H, Busse S, MÃ¼ller UJ, Mawrin C, Bernstein HG, Bogerts B, Steiner J. | Brain Behav Immun. 2014 Oct;41:59-64. doi: 10.1016/j.bbi.2014.05.012. Epub 2014 Jun 2. |  |  |  |  | 1 |  |  |  |
| Absence of neurodegeneration in the thalamus and caudate of elderly patients with schizophrenia | Falke E., Han L.-Y., Arnold S.E. | Psychiatry Research (2000) 93:2 (103-110). Date of Publication: 6 Mar 2000 |  |  |  |  | 1 |  |  |  |
| Astrocyte expression of D2-like dopamine receptors in the prefrontal cortex | Mladinov M., Mayer D., Brčić L., Wolstencroft E., Man N.T., Holt I., Hof P.R., Morris G.E., Šimić G. | Translational Neuroscience (2010) 1:3 (238-243). Date of Publication: September 2010 |  |  | 1 |  |  |  |  |  |
| Expression of immune genes on chromosome 6p21.3-22.1 in schizophrenia | Sinkus M.L., Adams C.E., Logel J., Freedman R., Leonard S. | Brain, Behavior, and Immunity (2013) 32 (51-62). Date of Publication: August 2013 | 1 |  |  |  |  |  |  |  |
| Inflammation-related genes up-regulated in schizophrenia brains | Saetre P., Emilsson L., Axelsson E., Kreuger J., Lindholm E., Jazin E. | BMC Psychiatry (2007) 7 Article Number: 46. Date of Publication: 6 Sep 2007 |  |  |  |  |  |  |  | 1 |
| Population-dependent contribution of the major histocompatibility complex region to schizophrenia susceptibility | Yamada K., Hattori E., Iwayama Y., Toyota T., Iwata Y., Suzuki K., Kikuchi M., Hashimoto T., Kanahara N., Mori N., Yoshikawa T. | Schizophrenia Research (2015) 168:1-2 (444-449) Article Number: 6506. Date of Publication: 1 Oct 2015 |  |  |  |  | 1 |  |  |  |
| Common mechanisms in neurodegeneration and neuroinflammation: a BrainNet Europe gene expression microarray study. | Durrenberger PF, [Fernando FS](https://www.ncbi.nlm.nih.gov/pubmed/?term=Fernando%20FS%5BAuthor%5D&cauthor=true&cauthor_uid=25119539), [Kashefi SN](https://www.ncbi.nlm.nih.gov/pubmed/?term=Kashefi%20SN%5BAuthor%5D&cauthor=true&cauthor_uid=25119539), [Bonnert TP](https://www.ncbi.nlm.nih.gov/pubmed/?term=Bonnert%20TP%5BAuthor%5D&cauthor=true&cauthor_uid=25119539), [Seilhean D](https://www.ncbi.nlm.nih.gov/pubmed/?term=Seilhean%20D%5BAuthor%5D&cauthor=true&cauthor_uid=25119539), [Nait-Oumesmar B](https://www.ncbi.nlm.nih.gov/pubmed/?term=Nait-Oumesmar%20B%5BAuthor%5D&cauthor=true&cauthor_uid=25119539), [Schmitt A](https://www.ncbi.nlm.nih.gov/pubmed/?term=Schmitt%20A%5BAuthor%5D&cauthor=true&cauthor_uid=25119539), [Gebicke-Haerter PJ](https://www.ncbi.nlm.nih.gov/pubmed/?term=Gebicke-Haerter%20PJ%5BAuthor%5D&cauthor=true&cauthor_uid=25119539), [Falkai P](https://www.ncbi.nlm.nih.gov/pubmed/?term=Falkai%20P%5BAuthor%5D&cauthor=true&cauthor_uid=25119539), [Grünblatt E](https://www.ncbi.nlm.nih.gov/pubmed/?term=Gr%C3%BCnblatt%20E%5BAuthor%5D&cauthor=true&cauthor_uid=25119539), [Palkovits M](https://www.ncbi.nlm.nih.gov/pubmed/?term=Palkovits%20M%5BAuthor%5D&cauthor=true&cauthor_uid=25119539), [Arzberger T](https://www.ncbi.nlm.nih.gov/pubmed/?term=Arzberger%20T%5BAuthor%5D&cauthor=true&cauthor_uid=25119539), [Kretzschmar H](https://www.ncbi.nlm.nih.gov/pubmed/?term=Kretzschmar%20H%5BAuthor%5D&cauthor=true&cauthor_uid=25119539), [Dexter DT](https://www.ncbi.nlm.nih.gov/pubmed/?term=Dexter%20DT%5BAuthor%5D&cauthor=true&cauthor_uid=25119539), [Reynolds R](https://www.ncbi.nlm.nih.gov/pubmed/?term=Reynolds%20R%5BAuthor%5D&cauthor=true&cauthor_uid=25119539). | J Neural Transm (Vienna). 2015 Jul;122(7):1055-68. doi: 10.1007/s00702-014-1293-0. Epub 2014 Aug 13. | 1 |  |  |  |  |  |  |  |
| Regulation of immune-modulatory genes in left superior temporal cortex of schizophrenia patients: a genome-wide microarray study. | Schmitt A, [Leonardi-Essmann F](https://www.ncbi.nlm.nih.gov/pubmed/?term=Leonardi-Essmann%20F%5BAuthor%5D&cauthor=true&cauthor_uid=21091092), [Durrenberger PF](https://www.ncbi.nlm.nih.gov/pubmed/?term=Durrenberger%20PF%5BAuthor%5D&cauthor=true&cauthor_uid=21091092), [Parlapani E](https://www.ncbi.nlm.nih.gov/pubmed/?term=Parlapani%20E%5BAuthor%5D&cauthor=true&cauthor_uid=21091092), [Schneider-Axmann T](https://www.ncbi.nlm.nih.gov/pubmed/?term=Schneider-Axmann%20T%5BAuthor%5D&cauthor=true&cauthor_uid=21091092), [Spanagel R](https://www.ncbi.nlm.nih.gov/pubmed/?term=Spanagel%20R%5BAuthor%5D&cauthor=true&cauthor_uid=21091092), [Arzberger T](https://www.ncbi.nlm.nih.gov/pubmed/?term=Arzberger%20T%5BAuthor%5D&cauthor=true&cauthor_uid=21091092), [Kretzschmar H](https://www.ncbi.nlm.nih.gov/pubmed/?term=Kretzschmar%20H%5BAuthor%5D&cauthor=true&cauthor_uid=21091092), [Herrera-Marschitz M](https://www.ncbi.nlm.nih.gov/pubmed/?term=Herrera-Marschitz%20M%5BAuthor%5D&cauthor=true&cauthor_uid=21091092), [Gruber O](https://www.ncbi.nlm.nih.gov/pubmed/?term=Gruber%20O%5BAuthor%5D&cauthor=true&cauthor_uid=21091092), [Reynolds R](https://www.ncbi.nlm.nih.gov/pubmed/?term=Reynolds%20R%5BAuthor%5D&cauthor=true&cauthor_uid=21091092), [Falkai P](https://www.ncbi.nlm.nih.gov/pubmed/?term=Falkai%20P%5BAuthor%5D&cauthor=true&cauthor_uid=21091092), [Gebicke-Haerter PJ](https://www.ncbi.nlm.nih.gov/pubmed/?term=Gebicke-Haerter%20PJ%5BAuthor%5D&cauthor=true&cauthor_uid=21091092). | World J Biol Psychiatry. 2011 Apr;12(3):201-15. doi: 10.3109/15622975.2010.530690. Epub 2010 Nov 22 | 1 |  |  |  |  |  |  |  |

**Supplementary Table 11. Qualitative assessment of studies included in meta-analysis microglial gene expression**

| First author | Year | Neuropathology^1^ | General confounders^2^ | Correction for age, gender, PMI^3^ | Extra confounders^4^ | Description methods^5^ | Diagnosis schizophrenia^6^ | Description demographics^7^ | Reporting Outcome^8^ |
| --- | --- | --- | --- | --- | --- | --- | --- | --- | --- |
| Nakatani | 2006 | - | + | c | + | + | +/- | + | +/- |
| Schmitt | 2010 | + | + | m, c | + | + | + | + | + |
| Sinkus | 2013 | + | + | m, c | + | + | +/- | + | + |
| Durrenberger | 2014 | + | + | m | + | + | + | +/- | + |
| Fiorentino | 2016 | - | + | - | + | + | +/- | + | +/- |
| Seredenina | 2017 | + | + | m, c | + | + | +/- | +/- | +/- |
| Lopez-Gonzalez | 2019 | + | + | m, c | + | + | + | +/- | +/- |
| *Description of qualitative categories:*  ^1^ Neuropathology: + donors checked for neuropathology and report thereof; - donors not checked for neuropathology or no report thereof.  ^2^ General confounders: + Reporting on age, sex and post-mortem interval (PMI) for SCZ and control groups; +/- 1 or 2 of these variables missing; - no information  ^3^ Matching/correction for confounders applied: m = matching applied; c = analysis of the impact of confounders and applied correction if necessary; - did not check for influence age and PMI  ^4^ Extra confounders: + additional confounders reported, such as medication use, pH, cause of death, ilness duration, age of onset; - no information  ^5^ Description methods: + Complete description of technical methodology and analyses (for replication purposes); +/- missing information; - no information  ^6^ Diagnosis schizophrenia: + Prospective based on structured interview or psychiatric assessment; +/- Retrospective based on charts; - No information reported  ^7^ Description demographics: + individual demographics given; +/- only provided on group level; - no information  ^8^ Reporting outcomes: + quantitative data reported; +/- value extraction from graphs required; - no values available for comparison microglia density SCZ vs control | | | | | | | | | |

|  |
| --- |

**Supplementary Table 12. Studies included in meta-analysis microglial gene expression.**

| First author | Year | Included in  Van Kesteren? | Brain bank* | N control | N SCZ | Age controls  (mean years) | Age SCZ  (mean years) | Sex control (male\|female) | Sex SCZ (male\|female) | PMI control  (mean hours) | PMD SCZ  (mean hours) | pH control | pH SCZ |
| --- | --- | --- | --- | --- | --- | --- | --- | --- | --- | --- | --- | --- | --- |
| Nakatani | 2006 | No | VIFM | 6 | 6 | 61.4 | 61.4 | 3 I 4 | 3 I 4 | 39.9 | 41.6 | 6.4 | 6.32 |
| Schmitt | 2011 | No | GOTT | 6 | 6 | 61.2 | 66.3 | 8 I 2 | 5 I 5 | 20.6 | 19.6 | 6.8 | 6.7 |
| Sinkus | 2013 | No | COL | 47 | 42 | 52.6 | 51.4 | 33 I 14 | 28 I 14 | 13 | 20.1 | 6.6 | 6.5 |
| Durrenberger | 2015 | No | GOTT | 6-8 | 8-9 | 61.2 | 66.3 | 5 I 5 | 5 I 5 | 17.3 | 20.8 | 6.4 | 6.5 |
| Fiorentino | 2016 | No | MAR | 14 | 10 | 33.3 | 44.2 | 15 I 0 | 15 I 0 | 16.7 | 18.1 | NR | NR |
| Seredenina | 2017 | No | SMRI | 30-33 | 31-33 | 48.1 | 44.2 | 9 \| 6 | 9 \| 6 | 23.7 | 33.7 | 6.3 | 6.1 |
| Lopez-Gonzalez | 2019 | No | BAR | 14 | 14 | 71 | 76 | 14 I 0 | 14 I 0 | 5.8 | 4.8 | 6.78 | 6.80 |
| * VIFM = Victorian Institute of Forensic Medicine; GOTT = Brain Bank for Psychiatric Diseases at the Gottingen University; COL = University of Colorado; MAR = Maryland Brain Bank;  BAR = HUB-ICO-IDIBELL Biobank Barcelona Spain;n SMRI = Stanley Medical Research Institute’s brain collection; NBB = Netherlands Brain Bank; EBB = Edinburgh Brain Bank | | | | | | | | | | | | | |

| **Supplementary Table 13. Outcomes studies included in meta-analysis microglial gene expression.**   \| First author \| Year \| n control \| n schizophrenia \| Brain region \| Marker used \| Method \| Expression mean control \| Expression STDEV control \| Expression mean SCZ \| Expression STDEV SCZ \| \| --- \| --- \| --- \| --- \| --- \| --- \| --- \| --- \| --- \| --- \| --- \| \| Nakatani \| 2006 \| 6 \| 6 \| DLPFC \| *HLA-DRA* \| qRT-PCR \| 0.49 \| 0.14 \| 0.40 \| 0.17 \| \| Schmitt \| 2011 \| 6 \| 6 \| Temporal cortex \| *HLA-DRB3* \| qRT-PCR \| 4.90 \| 4.00 \| 4.30 \| 1.20 \| \| Sinkus \| 2013 \| 47 \| 42 \| Hippocampus \| *HLA-DRA* \| qRT-PCR \| * \| * \| * \| * \| \| Durrenberger \| 2015 \| 8 \| 9 \| Temporal cortex \| *HLA-DRA* \| qRT-PCR \| 1.47 \| 1.72 \| 0.55 \| 0.38 \| \| Durrenberger \| 2015 \| 6 \| 8 \| Temporal cortex \| *HLA-DRB4* \| qRT-PCR \| 1.30 \| 0.67 \| 0.63 \| 0.16 \| \| Fiorentino \| 2016 \| 14 \| 10 \| Frontal cortex \| *AIF1* (Iba1) \| qRT-PCR \| 1.37 \| 1.05 \| 0.25 \| 0.18 \| \| Seredenina \| 2017 \| 30 \| 33 \| Prefrontal cortex \| *NOX2* \| qRT-PCR \| 0.32 \| 0.13 \| 0.32 \| 0.15 \| \| Seredenina \| 2017 \| 32 \| 31 \| Cingulate cortex \| *NOX2* \| qRT-PCR \| 0.74 \| 0.44 \| 0.67 \| 0.32 \| \| Seredenina \| 2017 \| 33 \| 32 \| Cingulate cortex \| *AIF1* (Iba1) \| qRT-PCR \| 0.62 \| 0.31 \| 0.49 \| 0.19 \| \| Seredenina \| 2017 \| 32 \| 31 \| Cingulate cortex \| *CD68* \| qRT-PCR \| 0.67 \| 0.34 \| 0.64 \| 0.30 \| \| Seredenina \| 2017 \| 33 \| 33 \| Cingulate cortex \| *ITGAM* (CD11b) \| qRT-PCR \| 0.64 \| 0.34 \| 0.56 \| 0.22 \| \| Lopez-Gonzalez \| 2019 \| 14 \| 14 \| DLPFC \| *CD68* \| qRT-PCR \| 1.74 \| 1.86 \| 0.57 \| 0.42 \| \| Lopez-Gonzalez \| 2019 \| 14 \| 14 \| DLPFC \| *AIF*1 (Iba1) \| qRT-PCR \| 1.38 \| 1.02 \| 0.96 \| 0.60 \| \| Lopez-Gonzalez \| 2019 \| 14 \| 14 \| DLPFC \| *CSF1R* \| qRT-PCR \| 2.09 \| 1.85 \| 0.85 \| 0.64 \| \| Lopez-Gonzalez \| 2019 \| 14 \| 14 \| DLPFC \| *TGFB1* \| qRT-PCR \| 1.16 \| 0.62 \| 0.99 \| 0.44 \| \| DLPFC = dorsolateral prefrontal cortex; qRT-PCR = quantitative real-time- polymerase chain reactions; STDEV = standard deviation; SCZ = schizophrenia * mean and STDEV were not given, used P-value 0.7973 and direction of effect was negative in meta-analyses. \| \| \| \| \| \| \| \| \| \| \| |
| --- | --- | --- | --- | --- | --- | --- | --- | --- | --- | --- | --- | --- | --- | --- | --- | --- | --- | --- | --- | --- | --- | --- | --- | --- | --- | --- | --- | --- | --- | --- | --- | --- | --- | --- | --- | --- | --- | --- | --- | --- | --- | --- | --- | --- | --- | --- | --- | --- | --- | --- | --- | --- | --- | --- | --- | --- | --- | --- | --- | --- | --- | --- | --- | --- | --- | --- | --- | --- | --- | --- | --- | --- | --- | --- | --- | --- | --- | --- | --- | --- | --- | --- | --- | --- | --- | --- | --- | --- | --- | --- | --- | --- | --- | --- | --- | --- | --- | --- | --- | --- | --- | --- | --- | --- | --- | --- | --- | --- | --- | --- | --- | --- | --- | --- | --- | --- | --- | --- | --- | --- | --- | --- | --- | --- | --- | --- | --- | --- | --- | --- | --- | --- | --- | --- | --- | --- | --- | --- | --- | --- | --- | --- | --- | --- | --- | --- | --- | --- | --- | --- | --- | --- | --- | --- | --- | --- | --- | --- | --- | --- | --- | --- | --- | --- | --- | --- | --- | --- | --- | --- | --- | --- | --- | --- | --- | --- | --- | --- | --- | --- | --- | --- | --- | --- | --- | --- | --- |
|  |

**Supplementary Table 14. Gene list of up and down-regulated genes in schizophrenia**

| Gene name | Log 2 fold change | FDR | Direction |
| --- | --- | --- | --- |
| *ACY3* | -0.527810256 | 5.91e-07 | Down |
| *ADAM28* | -0.23043082 | 0.000362305 | Down |
| *ADAP2* | -0.125485841 | 0.001615864 | Down |
| *AIF1* | -0.191755236 | 0.013902754 | Down |
| *AOAH* | -0.157329648 | 0.00037532 | Down |
| *APBB1IP* | -0.196511074 | 0.001089713 | Down |
| *ARHGAP25* | -0.095318565 | 0.01469453 | Down |
| *B3GNT5* | -0.116175968 | 0.017465484 | Down |
| *BIN2* | -0.21595858 | 2.81e-05 | Down |
| *BLNK* | -0.307083506 | 5.16e-06 | Down |
| *C3* | -0.174020018 | 0.028974787 | Down |
| *C3AR1* | -0.167102975 | 0.032985837 | Down |
| *CD33* | -0.195277606 | 0.003515369 | Down |
| *CD69* | -0.262743575 | 0.004563371 | Down |
| *CD84* | -0.289702808 | 1.03e-05 | Down |
| *CD86* | -0.170077252 | 0.024013179 | Down |
| *CLEC17A* | -0.327536666 | 0.00052149 | Down |
| *CLEC7A* | -0.230078742 | 0.000833164 | Down |
| *CLEC9A* | -0.295637048 | 0.000918088 | Down |
| *CMKLR1* | -0.11808515 | 0.011776256 | Down |
| *CSF1R* | -0.176016236 | 0.011831584 | Down |
| *CSF2RA* | -0.250545429 | 5.66e-06 | Down |
| *CX3CR1* | -0.464321935 | 9.88e-08 | Down |
| *CYBB* | -0.168850314 | 0.010124573 | Down |
| *CYTH4* | -0.143835844 | 0.014464278 | Down |
| *CYTL1* | -0.304822762 | 9.22e-06 | Down |
| *DHRS9* | -0.232102286 | 0.001713145 | Down |
| *DOCK2* | -0.11206826 | 0.023451564 | Down |
| *DOCK8* | -0.165847851 | 0.001185978 | Down |
| *FGL2* | -0.137772755 | 0.00923468 | Down |
| *GAL3ST4* | -0.154361044 | 0.000436557 | Down |
| *GIMAP2* | -0.108778713 | 0.015446716 | Down |
| *GIMAP4* | -0.104494476 | 0.004320026 | Down |
| *GPR183* | -0.328899728 | 1.56e-05 | Down |
| *GPR34* | -0.216028545 | 0.003052735 | Down |
| *HAVCR2* | -0.116272017 | 0.012842865 | Down |
| *HHEX* | -0.149926563 | 0.001153881 | Down |
| *HLA-DMB* | -0.216001512 | 0.001629283 | Down |
| *HPGDS* | -0.296021036 | 0.000129203 | Down |
| *IGSF6* | -0.166814529 | 0.006801042 | Down |
| *IKZF1* | -0.139282536 | 0.04271905 | Down |
| *IL18* | -0.196141568 | 0.001893388 | Down |
| *INPP5D* | -0.10651581 | 0.00545944 | Down |
| *IRF8* | -0.22942027 | 0.000971033 | Down |
| *ITGAM* | -0.165539682 | 0.004030505 | Down |
| *ITGAX* | -0.304906996 | 2.03e-08 | Down |
| *KCNQ1* | -0.146236103 | 0.012269468 | Down |
| *LILRB4* | -0.227797937 | 0.001598405 | Down |
| *LPAR5* | -0.291311203 | 6.29e-06 | Down |
| *LPAR6* | -0.229882157 | 5.41e-09 | Down |
| *LPXN* | -0.056826907 | 0.010022516 | Down |
| *LST1* | -0.211359328 | 0.000883982 | Down |
| *LY75* | -0.11181007 | 0.018990429 | Down |
| *LY86* | -0.231091792 | 0.001377254 | Down |
| *LYL1* | -0.156697292 | 0.000977999 | Down |
| *MILR1* | -0.208963113 | 0.013411269 | Down |
| *MPEG1* | -0.282188452 | 2.25e-06 | Down |
| *NCKAP1L* | -0.163890754 | 0.003429374 | Down |
| *OLFML3* | -0.210363229 | 6.42e-05 | Down |
| *OLR1* | -0.214748981 | 0.000152639 | Down |
| *P2RY12* | -0.415534178 | 4.67e-09 | Down |
| *P2RY13* | -0.353615316 | 1.93e-07 | Down |
| *PIK3R5* | -0.189374409 | 0.000302634 | Down |
| *PLCB2* | -0.114223942 | 0.001878157 | Down |
| *PLD4* | -0.327648738 | 4.74e-06 | Down |
| *PTAFR* | -0.22282364 | 0.000133644 | Down |
| *RASAL3* | -0.217432958 | 3.98e-05 | Down |
| *RGS10* | -0.212446059 | 9.11e-05 | Down |
| *SAMSN1* | -0.156458607 | 0.019439212 | Down |
| *SASH3* | -0.136833802 | 0.047731742 | Down |
| *SELPLG* | -0.369688147 | 4.62e-08 | Down |
| *SIGLEC11* | -0.272890533 | 0.000882146 | Down |
| *SLC37A2* | -0.132544925 | 0.009792501 | Down |
| *SLC7A7* | -0.129245825 | 0.03842788 | Down |
| *ST6GAL1* | -0.099086545 | 2.01e-05 | Down |
| *SUCNR1* | -0.444166772 | 1.38e-06 | Down |
| *SUSD3* | -0.333403239 | 2.41e-05 | Down |
| *SYK* | -0.19127978 | 0.003785714 | Down |
| *TAL1* | -0.181827222 | 7.97e-05 | Down |
| *TBXAS1* | -0.128010465 | 0.023620514 | Down |
| *TLR10* | -0.144470579 | 0.021689647 | Down |
| *TLR6* | -0.140434573 | 0.00732852 | Down |
| *TLR7* | -0.197356135 | 0.006567413 | Down |
| *TMEM106A* | -0.15959363 | 0.000295738 | Down |
| *TMEM119* | -0.23651205 | 0.000398798 | Down |
| *TNFAIP8L2* | -0.181694795 | 0.007278867 | Down |
| *TNFRSF13C* | -0.336155888 | 1.79e-05 | Down |
| *TRAF3IP3* | -0.173712315 | 4.63e-05 | Down |
| *TREM2* | -0.244846509 | 0.001329749 | Down |
| *WDFY4* | -0.179551087 | 3.82e-05 | Down |
| *CAPG* | 0.096437904 | 0.02626418 | Up |
| *FCGR3B* | 0.517594535 | 3.72e-07 | Up |
| *FPR1* | 0.246990572 | 0.022777853 | Up |
| *HAMP* | 0.331840247 | 0.013953499 | Up |
| *SERPINA1* | 0.218670577 | 0.020991979 | Up |
| *SLC11A1* | 0.354799282 | 3.05e-05 | Up |
| *TLR2* | 0.154362375 | 0.031315598 | Up |
| FDR = false discovery rate | | | |

**Supplementary Table 15. Gene expression of macrophage markers in postmortem brain tissue of schizophrenia patients**

|  |  | RNA-Sequencing (Gandal et al., 2018a) | | | Micro-array (Gandal et al., 2018b) | | |
| --- | --- | --- | --- | --- | --- | --- | --- |
| Marker | **Gene name** | **Log 2 fold change** | **p-value** | **FDR** | **Log 2 fold change** | **p-value** | **FDR** |
| CD169 | *SIGLEC1* | -0.016850385 | 0.77693688 | 0.890211393 | -0.004777157 | 0.735201906 | 0.863346664 |
| CD44 | *CD44* | 0.312375647 | 9.99E-07 | 4.12E-05 | 0.237644451 | 0.000578037 | 0.007685407 |
| CD49b | *ITGA4* | 0.072941452 | 0.020639972 | 0.088640786 | -0.011686799 | 0.54902355 | 0.742051153 |
| CCR2 | *CCR2* | NA | NA | NA | 0.00156672 | 0.940851136 | 0.971770758 |
| CD38 | *CD38* | -0.004231908 | 0.930575166 | 0.967476832 | 0.110369981 | 0.000205611 | 0.003716904 |
| CD274 | *PDL1* | 0.013749179 | 0.599275491 | 0.779001469 | NA | NA | NA |
| ITGAL | *CD11a* | -0.073259141 | 0.131566179 | 0.314538365 | 0.006638618 | 0.695075387 | 0.838177212 |
| FDR = false discovery rate.  *Gandal MJ, Zhang P, Hadjimichael E, Walker RL, Chen C, Liu S, Won H, Van Bakel H, Varghese M, Wang Y, Shieh AW, Haney J, Parhami S, Belmont J, Kim M, Losada PM, Khan Z, Mleczko J, Xia Y, Dai R, Wang D, Yang YT, Xu M, Fish K, Hof PR, Warrell J, Fitzgerald D, White K, Jaffe AE, Peters MA, Gerstein M, Liu C, Iakoucheva LM, Pinto D, Geschwind DH. (2018a). Transcriptome-wide isoform-level dysregulation in ASD, schizophrenia, and bipolar disorder. Science (80- ) 362.*  *Gandal MJ, Haney JR, Parikshak NN, Leppa V, Ramaswami G, Hartl C, Schork AJ, Appadurai V, Buil A, Werge TM, Liu C, White KP, Horvath S, Geschwind DH. (2018b). Shared molecular neuropathology across major psychiatric disorders parallels polygenic overlap. Science (80- ) 359:693–697.* | | | | | | | |
